# Serotonin 5-HT_6_ Receptor Ligands and Butyrylcholinesterase Inhibitors Displaying Antioxidant Activity—Design, Synthesis and Biological Evaluation of Multifunctional Agents against Alzheimer’s Disease

**DOI:** 10.3390/ijms23169443

**Published:** 2022-08-21

**Authors:** Krzysztof Więckowski, Natalia Szałaj, Beata Gryzło, Tomasz Wichur, Izabella Góral, Emilia Sługocka, Joanna Sniecikowska, Gniewomir Latacz, Agata Siwek, Justyna Godyń, Adam Bucki, Marcin Kołaczkowski, Anna Więckowska

**Affiliations:** 1 Department of Medicinal Chemistry, Faculty of Pharmacy, Jagiellonian University Medical College, 9 Medyczna St., 30-688 Kraków, Poland; 2 Department of Physicochemical Drug Analysis, Faculty of Pharmacy, Jagiellonian University Medical College, 9 Medyczna St., 30-688 Kraków, Poland; 3 Doctoral School of Medical and Health Sciences, Jagiellonian University Medical College, 16 Łazarza St., 31-530 Kraków, Poland; 4 Department of Technology and Biotechnology of Drugs, Faculty of Pharmacy, Jagiellonian University Medical College, 9 Medyczna St., 30-688 Kraków, Poland; 5 Department of Pharmacobiology, Faculty of Pharmacy Jagiellonian University Medical College, 9 Medyczna St., 30-688 Kraków, Poland

**Keywords:** Alzheimer’s disease, cholinesterase inhibitors, AChE, BuChE, 5-HT_6_ receptor ligands, antioxidant properties, metal-chelating properties, multifunctional ligands

## Abstract

Neurodegeneration leading to Alzheimer’s disease results from a complex interplay of a variety of processes including misfolding and aggregation of amyloid beta and tau proteins, neuroinflammation or oxidative stress. Therefore, to address more than one of these, drug discovery programmes focus on the development of multifunctional ligands, preferably with disease-modifying and symptoms-reducing potential. Following this idea, herein we present the design and synthesis of multifunctional ligands and biological evaluation of their 5-HT_6_ receptor affinity (radioligand binding assay), cholinesterase inhibitory activity (spectroscopic Ellman’s assay), antioxidant activity (ABTS assay) and metal-chelating properties, as well as a preliminary ADMET properties evaluation. Based on the results we selected compound **14** as a well-balanced and potent 5-HT_6_ receptor ligand (*K*_i_ = 22 nM) and human BuChE inhibitor (IC_50_ = 16 nM) with antioxidant potential expressed as a reduction of ABTS radicals by 35% (150 μM). The study also revealed additional metal-chelating properties of compounds **15** and **18**. The presented compounds modulating Alzheimer’s disease-related processes might be further developed as multifunctional ligands against the disease.

## 1. Introduction

Alzheimer’s disease (AD) is the most commonly diagnosed form of dementia with a significant impact on public health as well as the economy. According to the current data, more than 55 million people are living with dementia nowadays, and this number will almost triple by 2050 as a result of the demographic ageing phenomenon [1]. Patients suffering from AD manifest a gradual deterioration of cognitive functions and memory loss as well as accompanying behavioural impairments such as depression, psychosis, or anxiety. These symptoms arise from the emerging neurodegenerative changes within different neuron populations, especially cholinergic, serotonergic, and GABAergic. Among the factors that contribute to the development of AD, abnormal protein aggregates—amyloid-beta (Aβ) plaques and neurofibrillary tangles (NFTs) [2], oxidative stress, metal dyshomeostasis and inflammatory processes are of the utmost importance [3,4]. Their modulation may bring disease-modifying effects and can lead to effective therapeutics against AD.

Currently available first-line anti-AD drugs are acetyl- (AChE) and/or butyrylcholinesterase (BuChE) inhibitors that enhance disrupted cholinergic neurotransmission, temporarily relieving cognitive symptoms and slowing the disease progression [5]. This symptom-relieving function is essential in the treatment of patients with developed AD manifestations [6]. Therefore, many AD drug discovery programmes are based on the search for multifunctional ligands combining AChE/BuChE inhibitory activity with disease-modifying effects [7,8,9,10]. Although AChE initially appeared to be the key enzyme in acetylcholine metabolism, over the years it has become apparent that in AD BuChE takes over this function and its increased expression, especially in the hippocampus and temporal cortex, contributes largely to the development of the disease [11,12]. Not only does it decrease cholinergic neurotransmission but it also contributes to Aβ accumulation and aggregation, as shown in the 5XFAD/BChE-KO mice model (rapidly developing severe amyloid pathology BuChE-knockouts) where a fibrillar Aβ is significantly reduced [13].

A rather modest effect of cholinesterase inhibitors has been enhanced by the simultaneous use of 5-HT_6_ receptor (5-HT_6_R) antagonists. Idalopirdine and intepirdine demonstrated positive effects on cognition in phase II clinical trials as an adjunct to cholinesterase inhibitors in patients with moderate Alzheimer’s dementia [14,15]. 5-HT_6_ receptors are mainly distributed on neurons in the brain structures responsible for memory and learning—striatum, hippocampus and cerebral cortex—and are linked with multiple neurotransmitter systems including cholinergic, glutamatergic and GABAergic [16,17]. Its pharmacological blockade was shown to enhance neurotransmission and exert anxiolytic and antidepressant activity, thus alleviating behavioural and psychological symptoms in dementia [18,19]. Additionally, 5-HT_6_R antagonists may also have a disease-modulating effect by their influence on the reduction of Aβ (demonstrated in retrospective and prospective studies with SSRIs) [20] or modulation of synaptic plasticity [21], neuronal hyperexcitability and regulation of synaptic remodelling [22,23,24].

Oxidative stress plays a significant role in AD and other neurodegenerative diseases. It results from a disturbed balance between the production and accumulation of reactive oxygen species (ROS), leading to the progressive destruction of lipids, nucleic acids and proteins including enzymes involved in glucose metabolism [25]. Glucose dysmetabolism, energy deficiencies and resulting ions’ gradient disturbances lead to disrupted production and propagation of action potentials, and accumulation of free Ca^2+^ ions in cells contributing to neurons’ dysfunction and death [26]. Several mechanisms contribute to the development of oxidative stress, among them mitochondrial dysfunction, overexpression of oxidase enzymes, and imbalance of redox-active transition metal homeostasis [27,28]. A mounting body of evidence points to the crucial role of iron overload in the pathomechanism of AD. Increased concentrations of Fe^2+^, but also Cu^2+^, Al^3+^ and Zn^2+^ ions were observed post-mortem in AD patients’ brain samples and also in vivo [28,29,30,31] in amyloid plaques and the surrounding tissues. Increased levels of iron ions have been reported to have a multidirectional influence on the pathomechanism of AD by the generation of ROS, promotion of Aβ aggregation to oligomers and plaques, and aggregation of tau protein into neurofibrillary tangles [32]. Recently, this has received a lot of attention due to the definition of ferroptosis—a phenomenon of nonapoptotic programmed cell death driven by lethal lipid peroxidation resulting from an imbalance in redox homeostasis [33,34,35,36,37].

The complexity of AD etiopathogenesis and the variety of contributing factors makes it difficult to select biological targets whose modulation would bring beneficial and effective therapeutic results. However, it seems obvious that effective therapy of the disease needs to address the modulation of several factors involved in the development of the disease. In our research, we focused on the development of multifunctional ligands to address both the crucial processes involved in the development of AD and the symptoms resulting from neurodegeneration: antioxidant and metal-chelating activity combined with inhibition of cholinesterases and antagonism of the 5-HT_6_ receptors.

## 2. Results and Discussion

### 2.1. Design

**Figure 1:**
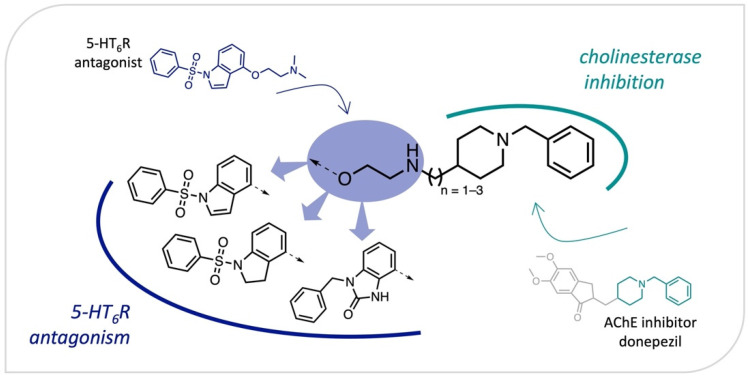
Design of new multifunctional ligands derived from AChE inhibitor—donepezil and 5-HT_6_ antagonists.

Following the idea of multifunctional ligands, we continue our research on 5-HT_6_R antagonists and cholinesterase inhibitors targeting processes underlying the pathomechanism of AD [38,39,40,41,42]. Previously, we have reported a series of indole-based compounds with antioxidant activity [42] and in the ongoing project, we selected three 2-aminoethoxy-substituted indole derivatives known for their 5-HT_6_R antagonistic properties—1-(phenylsulfonyl)-1*H*-indole, 1-(phenylsulfonyl)indoline and 1-benzyl-1,3-dihydro-2*H*-benzo[*d*]imidazol-2-one [40,43]—and connected them with the AChE-aiming 1-benzylpiperidine moiety via aliphatic linkers of different lengths (Figure 1). The 1-benzylpiperidine fragment is derived from the marketed acetylcholinesterase inhibitor donepezil and plays a crucial role in the interaction pattern of this drug with the active site of AChE [44,45]. In addition to AChE inhibitory activity, donepezil displayed antioxidant activity in vivo by reducing the brain concentrations of malondialdehyde in scopolamine and lipopolysaccharide models [46].

### 2.2. Synthesis

**Scheme 1:**
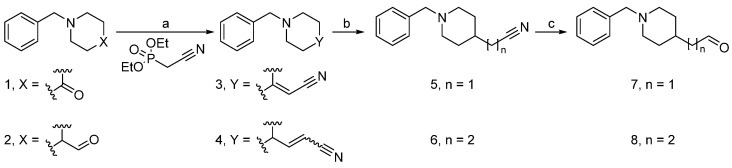
Synthesis of aldehydes **7** and **8**. Reagents and conditions: (**a**) NaH, DMF, 0 °C–rt, 12 h; (**b**) Mg/I_2_, MeOH, 0 °C–rt, 1h; (**c**) DIBAL-H, toluene, −90 °C, 1.5 h.

Key intermediates for the synthesis of the designed compounds were (1-benzylpiperidin-4-yl)alkyl aldehydes (**7** and **8**) and aryloxyethanamines (**9**–**11**). Aldehydes **7** and **8** were prepared according to a previously published method presented in Scheme 1 [47]. In this procedure, the Horner–Westworth–Emmons reaction between commercially available ketone **1** or aldehyde **2** and diethyl (cyanomethyl) phosphonate was used for the synthesis of acrylonitriles **3** and **4**. The olefins’ double bond was reduced with magnesium and I_2(cat.)_ in MeOH and the obtained alkanenitriles **5** and **6** were further reduced by DIBAL-H and hydrolysed to give the aldehydes **7** and **8** (Scheme 1).

**Scheme 2:**
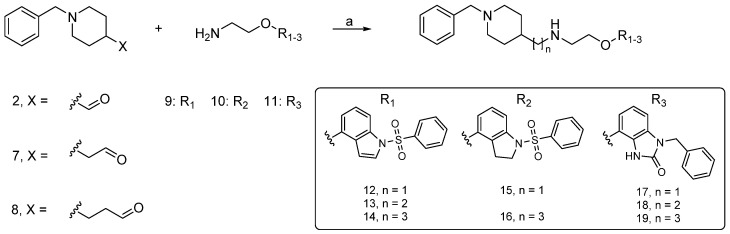
Synthesis of final compounds **12**–**19**. Reagents and conditions: (**a**) NaBH_3_CN, TEA, MeOH/THF 1:1, rt, 12 h.

We prepared aryloxyethanamines **9**–**11** according to the previously described procedures [40,48]. The final compounds **12**–**19** were obtained in the reductive amination reaction between the aldehydes **2** (commercially available), **7** or **8** and appropriate amines (**9**–**11**) in the presence of NaBH_3_CN as the reducing agent in the 1:1 mixture of methanol and THF (Scheme 2).

### 2.3. Biological Evaluation, SAR Analysis

*5-HT_6_R binding affinity and cholinesterase (AChE/BuChE) inhibitory activity*. We evaluated the pharmacological properties of the final compounds **12**–**19** in vitro according to the previously described, well-established protocols. Their affinities for recombinant human 5-HT_6_ receptors (*h*5-HT_6_R) were tested in a radioligand binding assay and the inhibitory potencies against human recombinant AChE (*h*AChE) and BuChE isolated from human plasma (*h*BuChE) in the spectrophotometric Ellman’s assay [38,49]. Table 1 lists the potencies expressed as *K*_i_ and IC_50_ values determined for the compounds and references: donepezil, tacrine and mianserin.

**Figure 2:**
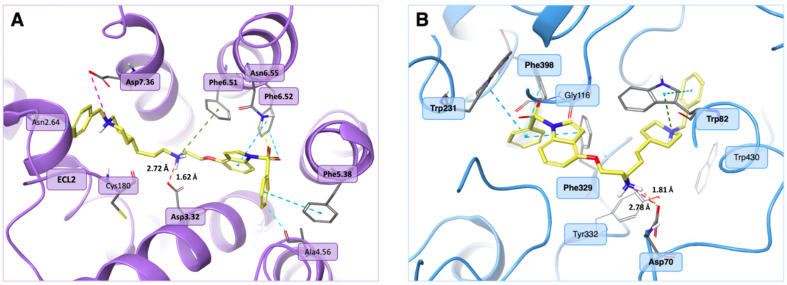
The predicted binding mode of compound **14** (yellow) in the site of the 5-HT_6_ receptor (**A**; a single conformation of the optimised homology model built on the 5-HT_1B_ template PDB 4iar, represented in violet; ECL2 residues were hidden for clarity) and *h*BuChE (**B**; a conformational model extracted from the MD simulation trajectory of PDB 7awh, with the criteria of frame selection being the presence of the most prevalent interactions indicated in the ligand–protein contacts analysis, represented in blue). Amino acid residues engaged in the ligand binding, thus forming H-bonds (dashed yellow lines), salt bridges (dashed pink lines), π-π/CH-π stacking interactions (dashed cyan lines) and cation-π interactions (dashed green lines) are presented as thick sticks. Distances between atoms forming H-bonds (**A** 1.62 Å; **B** 1.81 Å) and salt bridges (**A** 2.72 Å; **B** 2.78 Å) are presented beside (in **B** measured as average of the trajectory values with the formation cut-off 2.5 Å and 3.2 Å, respectively).

All the compounds displayed affinities for 5-HT_6_ receptors ranging from 17 nM to 598 nM. Molecular modelling studies on compound **14** disclosed that it adopts elongated conformation and a binding mode which is in line with the currently approved paradigm based on the published simulation on 5-HT_6_R homology models [50]. The interactions conserved for monoamine GPCRs-Asp3.32 salt bridge/H-bond and Phe6.52 CH-π stacking are the main anchoring forces. The orthosteric binding pocket interactions: CH-π stacking Phe5.38, Asn6.55 are unique for subtype 6 serotonin receptors. The salt bridge between the protonated amine and Asp7.36 has been described for tacrine-related 5-HT_6_R inhibitors (Figure 2) [38]. As we observed in our previous studies [38,40], the 1-(phenylsulfonyl)-1*H*-indole fragment, as in compounds **12**–**14**, ensures the highest potencies (17–22 nM).

**Figure 3:**
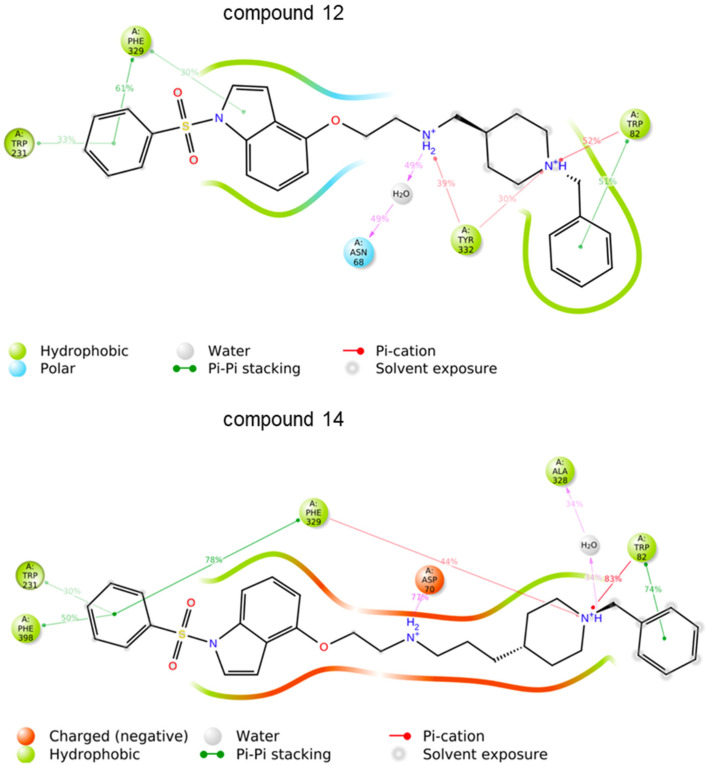
Ligand–protein interactions of compound **12** (up) and compound **14** (down) with BuChE. Notable dissimilarities regarding the pivotal salt bridge between Asp70 in the PAS and the secondary amine of the linker as well as the π-π stacking formed by Trp82 in the mid-gorge of BuChE are presented. The persistence of each contact is represented as the participation percentage of the whole simulation time, seen as a value above the arrow depicting particular interaction.

The tested compounds showed similar potencies against both *h*AChE and *h*BuChE, except for compound **14,** which displayed 60-fold higher potency against *h*BuChE (*h*AChE IC_50_ = 0.930 µM vs. *h*BuChE IC_50_ = 0.016 µM). The predicted binding mode of compound **14** in BuChE is presented in Figure 2. To investigate the exceptional potency of compound **14** and differences in the activity between the related compounds, we performed MD simulations in Desmond MD System (D. E. Shaw Research, New York, NY, 2020, Maestro-Desmond Interoperability Tools, Schrödinger, New York, NY, 2020-4 Release). Interactions lasting more than 30% of the simulation time were documented (Figure 3). The conformation of the molecules corresponds with the co-crystal structures of the related compounds [40]. Indole moiety in **14**, accountable for the recognition by the 5-HT_6_R, was sandwiched between the acyl loop and the Gly116/117 oxyanion hole, and its position was preserved through MD simulation. The compound was bound in the catalytic active site of *h*BuChE and formed stable interactions, crucial for the inhibitory activity of: (1) conserved π-π and cation-π interactions between Trp82 and the protonated piperidine moiety, maintained through the entire MD simulation; (2) the salt bridge and H-bond between Asp70 and the protonated secondary amine in the linker part of the molecule, being the major interactions in the peripheral anionic site PAS; (3) aromatic π-π stacking interactions with Trp231 and Phe329 in the acyl pocket, observed and described in co-crystal studies of propidium [51,52], contributing to the binding stabilisation.

SAR analysis showed that the longer the linker, the higher the potency against both *h*AChE and *h*BuChE. This observation is consistent in all the series with the difference mostly pronounced for **12** and **14** in terms of *h*BuChE inhibition (IC_50_ = 2.3 μM vs. 0.016 μM, respectively). The shorter linker (methylene vs. propylene) was responsible for compromised flexibility, causing the unfavourable shift of benzyl piperidine moiety, interruption, and permanent breakage of major binding interaction, which is a salt bridge with Asp70 (Figure 3).

*Antioxidant activity.* As mentioned before, oxidative stress is one of the components of AD pathomechanism and is characterised by an imbalance in the production of ROS. One of the mechanisms by which antioxidants reduce the concentration of ROS and therefore prevent or slow the cell damage caused by free radicals is scavenging them [53]. Thus, we tested the scavenging ability of compounds **12**–**19** in the spectrophotometric ABTS assay that we used before [54]. In this assay, the ability of compounds to scavenge ABTS^•^ radicals, which are generated from 2,2’-azino-bis(3-ethylbenzothiazoline-6-sulphonic acid) (ABTS), is measured. We tested the compounds at three concentrations: 30, 75 and 150 μM after 5, 30 and 60 min of incubation using Trolox, a water-soluble vitamin E analogue, as a reference (Table S1 in *Supporting information*).

**Figure 4:**
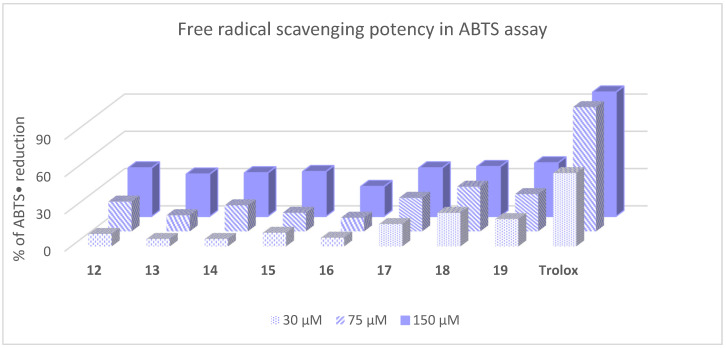
Results of ABTS assay: free radical scavenging potency of the tested compounds **12**–**19** and Trolox presented as percentage of ABTS^•^ reduction at 30, 75 and 150 µM after 60 min of incubation.

The results showed that the antioxidant capacity of these compounds depends on both incubation time and their concentration. After 60 min of incubation, all the compounds reduced ABTS^•^ by 6 to 27% at 30 µM, 11 to 36% at 75 µM and 24 to 43% at 150 µM (Figure 4), while Trolox reduced ABTS^•^ by 59% at 30 µM and 100% at higher concentrations. Almost all the compounds showed at least one-third of Trolox activity at 150 µM. Additionally, imidazoline-2-one-based compounds **17**–**19** kept this activity also at the lowest tested concentration. Antioxidant properties of the presented series of compounds, together with their other activities, may contribute to the prevention of cell damage in AD.

**Figure 5:**
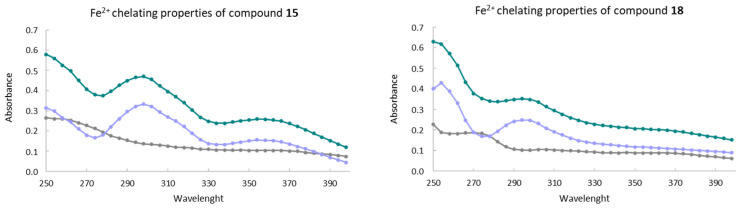
Fe^2+^ chelating properties of compounds **15** and **18** presented as a shift of absorbance (violet) between the absorbance of the ligand–Fe^2+^ mixture (green) and a sum of the absorbance of the ligand and Fe^2+^ (grey) at 50 µM concentration of ligand and Fe^2+^.

*Metal-chelating properties.* The homeostasis of metal ions in the brain is one of the important factors that determine its proper functioning. Disturbed levels of metal ions can lead to overproduction and aggregation of amyloid beta tau proteins and an increase in oxygen radicals production, leading to a degeneration of neurons observed in AD [55]. Additionally, it was observed that iron accumulation in microglia triggers a cascade of events leading to the production of a more sustained inflammatory response with increased reactive oxygen species (ROS) production and increased glycolysis [56,57]. Adjusting the balance of metal ions by supplementing or chelating may be beneficial in alleviating AD pathology. Thus, we tested the metal-chelating properties of compounds **12**–**19** against 10 metal ions, recognised as potentially involved in AD. The formation of ligand–ion (Al^3+^, Ca^2+^, Co^2+^, Cu^2+^, Fe^2+^, Fe^3+^, Mg^2+^, Ni^2+^, Pb^2+^ or Zn^2+^) complexes was measured spectrophotometrically (250–400 nm with 4 nm interval) at 50 µM concentrations of both, according to the protocol reported previously [54]. Metal-chelating ability was calculated as a shift of absorbance between the absorbance of the ligand–ion mixture and the sum of the absorbance of the ligand and ion separately. Compounds **15** and **18** were identified in the screening as Fe^2+^ ions chelators (Figure 5). This might be an advantageous feature of potential anti-neurodegenerative agents considering the correlation between iron overload, inflammation and glycolysis being inherent components of AD pathogenesis [58]. Therefore, the growing number of iron-chelating compounds with potential disease-modifying efficacy and the availability of MRI and CSF biomarkers of iron load encourage a deeper exploration of this therapeutic class in AD [59].

### 2.4. Preliminary In Vitro ADMET Studies

The search for biologically active substances should be accompanied by the assessment of their ADMET parameters, describing the absorption, distribution, metabolism, excretion and toxicity of novel compounds. Such an approach allows for the assessment of the potential of compounds for further development and minimises the risk of failure while progressing to in vivo testing. In our project, as a preliminary screening of ADMET parameters, we tested metabolic stability, interaction with cytochrome P450 isoenzymes (CYP3A4 and CYP2D6) and potential hepatotoxicity of the selected compounds **14**, **16** and **19** (each with a different 5-HT_6_-aiming fragment).

*Metabolic stability.* As the liver is the primary site of drug metabolism, we assessed the in vitro metabolic stability of compounds **14**, **16** and **19** on human and mouse liver microsomes (HLMs, MLMs). The compounds and verapamil, used as a reference, were incubated with HLMs and MLMs for 2 h and then the resulting mixtures were analysed with UPLC-MS. The compounds showed high stability, with at least 75% of each left in the unaffected form after incubation, while the figure with verapamil was ca. 27% (Table 2). Based on the analysis of the MS spectra of the metabolites supported by the in silico predictions by MetaSite 8.0.1, we concluded that the main metabolic pathway was oxidative deamination, taking place on the nitrogen atom either in the oxyethylamine moiety (comp. **14**) or in the piperidine ring (compounds **16** and **19**). For detailed results, including UPLC chromatograms and MS spectra, see the *Supporting information* (Figures S1–S16).

**Figure 6:**
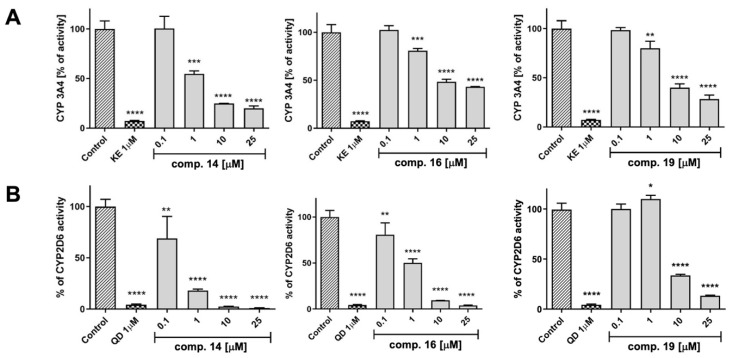
The influence of increasing concentrations of compounds **14**, **16** and **19** (0.1, 1, 10 and 25 µM) and references (KE, ketoconazole; QD, quinidine; both at 1 μM) on CYP3A4 (**A**) and CYP2D6 (**B**) activity. Statistical significance was evaluated in Graph Pad Prism 8.0.1 software using one-way ANOVA and Bonferroni’s multiple comparison post-test (**** *p* < 0.0001, *** *p* < 0.001, ** *p* < 0.01, * p < 0.05). The compounds were examined in triplicate.

*Influence on CYP3A4 and CYP2D6 activity.* Possible drug–drug interactions (DDIs) are an important issue for elderly patients, who are usually on multiple medications. They may result, among other things, from the interactions (inhibition or activation) of the cytochrome P450 isoenzymes, especially CYP3A4 and CYP2D6, which play the most significant role in drugs’ biotransformation [60]. Therefore, to predict the potential influence of the studied compounds (**14**, **16** and **19**) on the metabolism of other drugs, we used the CYP450 inhibition luminescence assay (Promega). None of the compounds showed any effect on CYP3A4 at 0.1 µM concentration. We observed inhibition of the enzyme starting from 1 µM concentration but it is worth noting that in all the concentrations (1, 10 and 25 µM) it was significantly lower than that of 1 µM of ketoconazole, used as a reference in the test (Figure 6A). The more pronounced effect of the compounds was observed for the CYP2D6 isoform. While at the concentration of 0.1 µM, compound **19** did not influence the activity of CYP2D6, compounds **14** and **16** inhibited the enzyme by ca. 20% (Figure 6B). The effect increased with the increasing concentrations of the compounds, with one exception: compound **19** at 1 µM showed a CYP2D6-activating effect. Nevertheless, none of the compounds was nearly as potent an inhibitor as quinidine, a drug used here as a reference.

**Figure 7:**
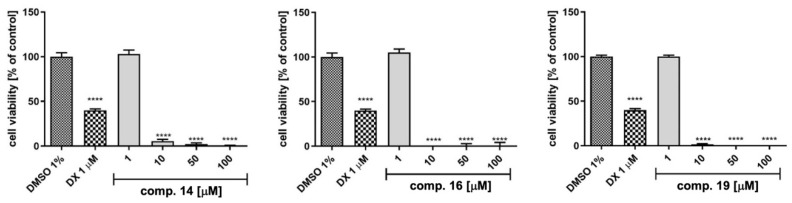
The effect of cytostatic drug doxorubicin (DX) and compounds **14**, **16** and **19** on *hepatoma* HepG2 cell lines’ viability after 72 h of incubation at 37 °C, 5% CO_2_. Statistical significance (**** *p* < 0.0001) was analysed by Graph Pad Prism 8.0.1 software using One-way ANOVA and Bonferroni’s multiple comparison post-test. The compounds were examined in quadruplicate.

*Hepatotoxicity.* To further investigate the safety of the compounds, we determined their hepatotoxicity. We used the MTS test that determines cell viability to assess the effect of compounds **14**, **16** and **19** on HepG2 (human hepatocellular carcinoma) cells [61]. The compounds were tested at 1, 10, 50 and 100 μM, and cytostatic drug doxorubicin (DX) at 1 μM was used as a reference compound. While none of the tested compounds showed a cytotoxic effect at the concentration of 1 μM, they caused a significant reduction in cell viability at higher concentrations (10–100 µM, Figure 7). For a more precise evaluation of the effect, we determined the IC_50_ value for compound **14,** which equalled 2.16 μM. This was a concentration at least 98-fold higher than the *K*_i_ for 5-HT_6_R and IC_50_ for *h*BuChE, which indicates a broad safety margin for the compound.

## 3. Materials and Methods

### 3.1. Chemistry

Commercially available reagents were purchased from Merck, Aldrich, Acros or ChemPur and were used without additional purification, except for triethylamine (TEA), which was purified by vacuum distillation. Anhydrous solvents were obtained by drying over sodium (THF) or CaH_2_ (DCM) followed by distillation under argon immediately before use. Microwave irradiation was carried out using the Discover LabMate reactor (CEM Corporation, USA). Reactions were monitored by thin-layer chromatography (TLC) (aluminium sheets precoated with silica gel 60 F_254_ (Merck)). The spots were visualised with UV light (254 nm) and by staining with a 0.5% solution of ninhydrin in propan-1-ol or a solution of 5% (NH_4_)_6_Mo_7_O_24_, 0.2% Ce(SO_4_)_2_, and 5% H_2_SO_4_ in water. Purification by column chromatography was carried out using silica gel mesh 0.063–0.200 mm (Sigma-Aldrich) as the stationary phase.

^1^H NMR and ^13^C NMR spectra were recorded on JEOL ECA400II or ECX500 at magnetic field strengths of 11.75 T, corresponding to ^1^H and ^13^C resonance frequencies of 500.16 MHz and 125.77 MHz at ambient temperature (25 °C). Chemical shifts (δ) are reported in parts per million (ppm) and referenced to the residual solvent signals (CDCl_3_ ^1^H: 7.26 ppm, ^13^C: 77.16 ppm; CD_3_OD ^1^H: 3.31 ppm, ^13^C: 49.00 ppm). Coupling constants (*J*) are reported in hertz (Hz). The purity and identity of the final compounds were confirmed with an ultra-performance liquid chromatography instrument (Waters ACQUITY equipped with a UPLC BEH C18 column, 1.7 μm, 2.1 100 mm; Acquity (10 min. gradient MeCN/H_2_O + 0.1% HCOOH, Q = 0.3 mL/min,) coupled to a Waters TQD mass spectrometer (ESI-tandem quadrupole) (Waters, Milford, MA, USA)).

#### 3.1.1. Previously Reported Compounds 

The following compounds were synthesized following previously published procedures:

2-((1-(Phenylsulfonyl)-1*H*-indol-4-yl) oxy) ethan-1-amine (**9**) [40,48]

2-((1-(Phenylsulfonyl) indolin-4-yl) oxy) ethan-1-amine (**10**) [40]

2-((1-Benzyl-2-oxo-2,3-dihydro-1*H*-benzo[d] imidazole-4-yl)oxy)ethan-1-aminium chloride (**11**) [40].

#### 3.1.2. General Procedure for the Synthesis of Nitriles **3** and **4** (GP1)

To a solution of NaH (60% dispersion in mineral oil, 1.1 eq.) in dry DMF, diethyl(cyanomethyl)phosphonate (1.1 eq.) was added dropwise under argon atmosphere at 0 °C and the mixture was stirred for 20 min at 0 °C. Then, a solution of carbonyl compound **1** or **2** (1 eq.) in dry DMF at 0 °C was added. The resulting mixture was warmed up to room temperature and stirred overnight. The reaction was quenched by adding water and extracted with Et_2_O three times. The organic extracts were combined, dried with anhydrous MgSO_4_, filtered and concentrated in a vacuum. The crude product was purified by column chromatography (DCM/EtOAc, 3:1).

##### 2-(1-Benzylpiperidin-4-ylidene)acetonitrile (**3**)

Following GP1, compound **3** was prepared using NaH (960 mg, 60% dispersion in mineral oil, 24 mmol), diethyl(cyanomethyl)phosphonate (3.88 mL, 24 mmol), 1-benzylpiperidin-4-one (**1**) (4.124 g, 3.89 mL, 21.8 mmol) in 42 mL of dry DMF (32 + 10 mL). Yield: 2.5 g (80%), orange oil. ^1^H NMR (500 MHz, CDCl_3_) δ 7.24–7.32 (m, 5H), 5.08 (s, 1H), 3.53 (s, 2H), 2.49–2.61 (m, 6H), 2.35-2.36 (t, J = 5.1 Hz, 2H). Formula: C_14_H_16_N_2_.

##### 3-(1-Benzylpiperidin-4-yl)acrylonitrile (**4**)

Following GP1, compound **4** was prepared using NaH (656 mg, 60% dispersion in mineral oil, 16.4 mmol), diethyl(cyanomethyl)phosphonate (2.90 mL, 16.4 mmol), 1-benzylpiperidine-4-carbaldehyde (**2**) (3.00 g, 2.94 mL, 14.77 mmol) in 29 mL of dry DMF (22 + 7 mL). Yield: 1.44 g (44%), pale yellow oil. ^1^H NMR (500 MHz, CDCl_3_) δ 7.20–7.35 (m, 5H), 6.31 (t, *J* = 10.3 Hz, 1H), 5.24 (d, *J* =11.0 Hz, 1H), 3.51 (s, 2H), 2.90 (d, *J* = 11.7 Hz, 2H), 2.51–2.70 (m, 1H), 1.94–2.15 (m, 2H), 1.63–1.76 (m, 2H), 1.41–1.60 (m, 2H). Formula: C_15_H_18_N_2_.

#### 3.1.3. General Procedure for the Synthesis of Nitriles **5** and **6** (GP2)

To a solution of nitrile **3** or **4** (1 eq.) in MeOH, Mg (20 eq.) and a catalytic amount of I_2_ were added and the mixture was sonicated in an ultrasonic bath until the nitrile was consumed (TLC). After the conversion, the reaction mixture was cooled in an ice bath and conc. HCl was added. When all material was dissolved, the mixture was neutralised to pH = 8, by adding 30% NaOH. The mixture was then extracted with EtOAc (3 × 50 mL) and the extracts were combined and dried over Na_2_SO_4_, filtered and concentrated under vacuum. The product was used in the next step without further purification.

##### 2-(1-Benzylpiperidin-4-yl)acetonitrile (**5**)

Following GP2, compound **5** was prepared using 2-(1-benzylpiperidin-4-ylidene)acetonitrile (**3**) (1.633 g, 7.7 mmol), Mg (3.742 g, 154 mmol), catalytic amount of I_2_ in 35 mL MeOH. Yield: 1.352 g (82%), yellow oil. ^1^H NMR (500 MHz, CDCl_3_) δ 7.24–7.34 (m, 5H), 3.55 (s, 2H), 2.93 (d, *J* = 11.8 Hz, 2H), 2.28 (d, *J* = 6.60 Hz, 2H), 2.00 (t, *J* = 52.77 Hz, 2H), 1.78 (m, 2H), 1.68 (m, 1H), 1.48 (m, 2H). Formula: C_14_H_18_N_2_.

##### 3-(1-Benzylpiperidin-4-yl)propanenitrile (**6**)

Following GP2, compound **6** was prepared using 3-(1-benzylpiperidin-4-yl)acrylonitrile (**4**) (1.741 g, 7.7 mmol), Mg (3.742 g, 154 mmol), catalytic amount of I_2_ in 35 mL MeOH. Yield: 1.510 g (87%), yellow oil. ^1^H NMR (500 MHz, CDCl_3_) δ 7.19–7.37 (m, 5 H), 3.50 (s, 2 H), 2.89 (d, *J* = 11.8 Hz, 2 H), 2.35 (t, *J* = 7.3 Hz, 2 H), 1.96 (td, *J* = 11.5, 1.9 Hz, 2 H), 1.54–1.73 (m, 4 H), 1.17–1.52 (m, 3 H). Formula: C_15_H_20_N_2_.

#### 3.1.4. General Procedure for the Synthesis of Aldehydes **7** and **8** (GP3)

The solution of nitrile **5** or **6** (1 eq.) in dry toluene in an oven-dried and argon-purged flask was cooled to –78 °C, and 1 M DIBAL-H solution in toluene (2 eq.) was added dropwise. The reaction mixture was stirred at –78 °C for 1.5 h. Then, MeOH was added, the solution was poured into 5% H_2_SO_4_ (aq) and finally basified with conc. NH_3_ (aq). The precipitate was filtered off and washed with EtOAc. The filtrate was extracted with AcOEt three times. The combined organic extracts were washed with brine, dried with anhydrous MgSO_4_ and purified by column chromatography (hexane/EtOAc, 2:1).

##### 2-(1-Benzylpiperidin-4-yl)acetaldehyde (**7**)

Following GP3, compound **7** was prepared using 2-(1-benzylpiperidin-4-yl)acetonitrile (**5**) (1 g, 4.71 mmol) and 9.42 mL of 1 M DIBAL-H solution in toluene in 20 mL of dry toluene. Yield: 0.897 g (88%), orange oil. ^1^H NMR (500 MHz, CDCl_3_) δ 9.73 (s, 1H), 7.29 (s, 5H), 3.48 (s, 2H), 2.85 (br d, *J* = 12.0 Hz, 2H), 2.37–2.23 (m, 2H), 1.99 (dt, *J* = 2.3, 12.0 Hz, 2H), 1.91–1.79 (m, 1H), 1.66 (br d, *J* = 13.2 Hz, 2H), 1.41–1.28 (m, 2H). Formula: C_14_H_19_NO.

##### 3-(1-Benzylpiperidin-4-yl)propanal (**8**)

Following GP3, compound **8** was prepared using 3-(1-benzylpiperidin-4-yl)propanenitrile (**6**) (1 g, 4.39 mmol) and 8.78 mL of 1 M DIBAL-H solution in toluene in 20 mL of dry toluene. Yield: 0.840 g (83%), colourless oil. ^1^H NMR (500 MHz, CDCl_3_) δ 9.76 (t, *J* = 1.6 Hz, 1H), 7.19–7.38 (m, 5H), 3.44–3.53 (m, 2H), 2.87 (d, *J* = 11.1 Hz, 2H), 2.43 (td, *J* = 7.5, 1.6 Hz, 2H), 1.92 (t, *J* = 11.8 Hz, 2H), 1.52–1.72 (m, 4H), 1.16–1.35 (m, 3H). Formula: C_15_H_21_NO.

#### 3.1.5. General Procedure for the Synthesis of Compounds **12**–**19** (GP4)

To a solution of an appropriate aldehyde (**2**, **7** or **8**) (1 eq.), amine as a free base or in form of hydrochloride (**9**–**11**) (1.3 eq.) and triethylamine (2.6 eq. or 5.12 eq. for hydrochlorides) in a mixture of THF/MeOH (1:1), NaBH_3_CN (3 or 5 eq.) was added. The mixture was stirred overnight at room temperature. After completion of the reaction, the solvents were evaporated, water was added and the solution was extracted with a mixture of CHCl_3_/isopropanol 3:1. The organic extracts were combined, washed with brine, dried with anhydrous MgSO_4_ and concentrated under reduced pressure. The crude product was purified by silica gel chromatography (EtOAc/MeOH/NH_3_(aq) 95:5:1) to give the final compound.

##### *N*-((1-Benzylpiperidin-4-yl)methyl)-2-((1-(phenylsulfonyl)-1*H*-indol-4-yl)oxy)ethan-1-amine (**12**)

Following GP4, compound **12** was prepared using 1-benzylpiperidine-4-carbaldehyde (**2**) (75 mg, 0.375 mmol), 2-((1-(phenylsulfonyl)-1*H*-indol-4-yl)oxy)ethan-1-amine (**9**) (158 mg, 0.5 mmol), triethylamine (113.83 mg, 156 ml, 1.125 mmol), NaBH_3_CN (70 mg, 1.125 mmol) in 4 mL of a mixture of THF/MeOH (1:1). Yield: 79 mg (42%), white solid. ^1^H NMR (500 MHz, CDCl_3_) δ 7.87 (dd, *J* = 1.2, 8.6 Hz, 2H), 7.60 (d, *J* = 8.6 Hz, 1H), 7.53 (t, *J* = 7.5 Hz, 1H), 7.48 (d, *J* = 3.7 Hz, 1H), 7.44 (t, *J* = 8.0 Hz, 2H), 7.32 (d, *J* = 4.6 Hz, 4H), 7.25–7.26 (m, 1H), 7.22 (t, *J* = 8.2 Hz, 1H), 6.76 (dd, *J* = 0.9, 3.7 Hz, 1H), 6.66 (d, *J* = 7.7 Hz, 1H), 4.16 (t, *J* = 5.2 Hz, 2H), 3.51 (s, 2H), 3.03 (t, *J* = 5.3 Hz, 2H), 2.91 (d, *J* = 11.5 Hz, 2H), 2.58 (d, *J* = 6.9 Hz, 2H), 1.95 (t, *J* = 10.9 Hz, 2H), 1.71 (dd, *J* = 1.0, 12.7 Hz, 2H), 1.48 (td, *J* = 4.0, 7.5 Hz, 1H), 1.23–1.37 (m, 3H). ^13^C NMR (126 MHz, CDCl_3_) δ 152.4, 138.3, 136.2, 133.9, 129.4 (2C), 129.3 (2C), 128.3 (2C), 127.1 (2C), 126.8 (2C), 125.8, 124.9, 121.3, 106.7, 106.3, 104.6, 67.7, 63.5, 55.9, 53.7, 49.0 (2C), 36.2, 30.5, 30.5. Formula: C_29_H_33_N_3_O_3_S; LC-MS: *m/z* 504 (M+H^+^).

##### 2-(1-Benzylpiperidin-4-yl)-*N*-(2-((1-(phenylsulfonyl)-1*H*-indol-4-yl) oxy)ethyl)ethan-1-amine (**13**)

Following GP4, compound **13** was prepared using 2-(1-benzylpiperidin-4-yl)acetaldehyde (**7**) (77 mg, 0.375), 2-((1-(phenylsulfonyl)-1*H*-indol-4-yl)oxy)ethan-1-amine (**9**) (158 mg, 0.5 mmol), triethylamine (113.83 mg, 156 ml, 1.125 mmol), NaBH_3_CN (70 mg, 1.125 mmol) in 4 mL of a mixture of THF/MeOH (1:1). Yield: 78 mg (40%), colourless oil. ^1^H NMR (500 MHz, CDCl_3_) δ 7.87–7.83 (m, 2H), 7.59 (d, *J* = 8.6 Hz, 1H), 7.52–7.48 (m, 1H), 7.46 (d, *J* = 4.0 Hz, 1H), 7.43–7.38 (m, 2H), 7.30 (d, *J* = 4.6 Hz, 4H), 7.27–7.23 (m, 1H), 7.20 (t, *J* = 8.0 Hz, 1H), 6.76 (d, *J* = 3.4 Hz, 1H), 6.64 (d, *J* = 8.0 Hz, 1H), 4.14 (t, *J* = 5.2 Hz, 2H), 3.48 (s, 2H), 3.02 (t, *J* = 5.2 Hz, 2H), 2.85 (br d, *J* = 12.0 Hz, 2H), 2.69 (t, *J* = 7.4 Hz, 2H), 2.05 (br s, 1H), 1.92 (br t, *J* = 11.5 Hz, 2H), 1.66–1.57 (m, 2H), 1.49–1.40 (m, 2H), 1.34–1.21 (m, 3H). ^13^C NMR (126 MHz, CDCl_3_) δ 152.3, 138.2, 136.1, 133.7, 129.2 (2C), 128.1 (2C), 126.9 (2C), 126.7 (2C), 125.6 (2C), 124.7, 121.2, 106.6, 106.6, 106.2, 104.5, 67.5, 63.4, 53.7, 48.7 (2C), 47.2, 36.8 (2C), 33.7, 32.2. Formula: C_30_H_35_N_3_O_3_S; LC-MS: *m*/z 518 (M+H^+^).

##### 3-(1-Benzylpiperidin-4-yl)-*N*-(2-((1-(phenylsulfonyl)-1*H*-indol-4-yl)oxy)ethyl)propan-1-amine (**14**)

Following GP4, compound **14** was prepared using 3-(1-benzylpiperidin-4-yl)propanal (**8**) (87 mg, 0,375 mmol), 2-((1-(phenylsulfonyl)-1*H*-indol-4-yl)oxy)ethan-1-amine (**9**) (158 mg, 0.5 mmol), triethylamine (113.83 mg, 156 ml, 1.125 mmol), NaBH_3_CN (70 mg, 1.125 mmol) in 5 mL of a mixture of THF/MeOH (1:1). Yield: 60 mg (30%), white solid. ^1^H NMR (500 MHz, CDCl_3_) δ 7.89–7.84 (m, 2H), 7.61 (d, *J* = 8.0 Hz, 1H), 7.56–7.49 (m, 1H), 7.48 (d, *J* = 4.0 Hz, 1H), 7.47–7.39 (m, 2H), 7.34–7.29 (m, 4H), 7.28–7.24 (m, 1H), 7.22 (t, *J* = 8.3 Hz, 1H), 6.81–6.76 (m, 1H), 6.68–6.63 (m, 1H), 4.17 (t, *J* = 5.2 Hz, 2H), 3.50 (s, 2H), 3.04 (t, *J* = 5.4 Hz, 2H), 2.88 (br d, *J* = 11.5 Hz, 2H), 2.71–2.62 (m, 2H), 2.18 (br d, *J* = 2.3 Hz, 1H), 1.97–1.88 (m, 2H), 1.65 (br d, *J* = 9.7 Hz, 2H), 1.57–1.45 (m, 2H), 1.31–1.20 (m, 5H). ^13^C NMR (126 MHz, CDCl_3_) δ 152.4, 138.3, 136.3, 133.9, 129.4 (2C), 129.3 (2C), 128.2 (2C), 127.0, 126.8 (2C), 125.8 (2C), 124.9, 121.3, 106.7, 106.4, 104.6, 67.7, 63.6, 53.9, 50.2, 48.8 (2C), 35.8 (2C), 34.3, 32.4, 27.5. Formula: C_31_H_37_N_3_O_3_S; LC-MS: *m/z* 532 (M + H^+^).

##### *N*-((1-Benzylpiperidin-4-yl) methyl)-2-((1-(phenylsulfonyl) indolin-4-yl)oxy)ethan-1-amine (**15**)

Following GP4, compound **15** was prepared using 1-benzylpiperidine-4-carbaldehyde (**2**) (76 mg, 0.375 mmol), 2-((1-(phenylsulfonyl)indolin-4-yl)oxy)ethan-1-amine (**10**) (134 mg, 0.5 mmol), triethylamine (113.83 mg, 156 ml, 1.125 mmol), NaBH_3_CN (128 mg, 1.875 mmol) in 5 mL of a mixture of THF/MeOH (1:1). Yield: 53 mg (31%), white solid. ^1^H NMR (500 MHz, CDCl_3_) δ 7.86–7.76 (m, 2H), 7.63–7.49 (m, 1H), 7.47–7.42 (m, 2H), 7.34–7.23 (m, 6H), 7.14 (t, *J* = 8.3 Hz, 1H), 6.51 (d, *J* = 8.0 Hz, 1H), 4.04 (t, *J* = 5.2 Hz, 2H), 3.93 (t, *J* = 8.3 Hz, 2H), 3.54 (s, 2H), 2.94 (t, *J* = 5.2 Hz, 3H), 2.82 (t, *J* = 8.6 Hz, 2H), 2.54 (d, *J* = 6.9 Hz, 2H), 2.05–1.92 (m, 2H), 1.73–1.65 (m, 2H), 1.47 (dtd, *J* = 3.7, 7.2, 11.0 Hz, 1H), 1.35–1.23 (m, 4H). ^13^C NMR (126 MHz, CDCl_3_) δ 155.4, 143.4, 137.3, 133.2, 129.5 (2C), 129.3 (2C), 129.1 (2C), 128.3 (2C), 127.4 (2C), 119.2 (2C), 108.1, 107.0, 67.3, 63.2, 55.6, 53.4, 50.3, 48.7 (2C), 35.9 (2C), 30.2, 25.0. Formula: C_29_H_35_N_3_O_3_S; LC-MS: *m/z* 506 (M + H^+^).

##### 3-(1-Benzylpiperidin-4-yl)-*N*-(2-((1-(phenylsulfonyl) indolin-4-yl)oxy)ethyl)propan-1-amine (**16**)

Following GP4, compound **16** was prepared using 3-(1-benzylpiperidin-4-yl)propanal (**8**) (87 mg, 0.375 mmol), 2-((1-(phenylsulfonyl)indolin-4-yl)oxy)ethan-1-amine (**10**) (159 mg, 0.5 mmol), triethylamine (113.83 mg, 156 ml, 1.125 mmol), NaBH_3_CN (128 mg, 1.875 mmol) in 5 mL of a mixture of THF/MeOH (1:1). Yield: 70 mg (35%), white solid. ^1^H NMR (500 MHz, CDCl_3_) δ 7.81–7.76 (m, 2H), 7.57–7.49 (m, 1H), 7.47–7.39 (m, 2H), 7.32–7.29 (m, 4H), 7.29–7.23 (m, 3H), 7.12 (t, *J* = 8.3 Hz, 1H), 6.49 (d, *J* = 8.0 Hz, 1H), 4.19 (br d, *J* = 5.2 Hz, 1H), 4.03 (t, *J* = 5.4 Hz, 2H), 3.93–3.88 (m, 2H), 3.56 (s, 2H), 2.98–2.90 (m, 4H), 2.82 (t, *J* = 8.6 Hz, 2H), 2.65–2.60 (m, 2H), 2.02–1.95 (m, 2H), 1.62 (br d, *J* = 11.5 Hz, 2H), 1.52–1.45 (m, 2H), 1.25–1.21 (m, 4H). ^13^C NMR (126 MHz, CDCl_3_) δ 155.2, 143.4, 137.0, 133.2, 129.8 (2C), 129.3 (2C), 129.2, 128.5 (2C), 128.4 (2C), 127.4, 119.2, 108.0, 107.8, 107.0, 66.9, 62.9, 53.4, 50.3, 49.6, 48.2 (2C), 35.4 (2C), 33.9, 31.7, 26.7, 25.0. Formula: C_31_H_39_N_3_O_3_S; LC-MS: *m/z* 534 (M + H^+^).

##### 1-Benzyl-4-(2-(((1-benzylpiperidin-4-yl)methyl)amino)ethoxy)-1,3-dihydro-2*H*-benzo[d]imidazol-2-one (**17**)

Following GP4, compound **17** was prepared using 1-benzylpiperidine-4-carbaldehyde (**2**) (76 mg, 0.357 mmol), 2-((1-benzyl-2-oxo-2,3-dihydro-1*H*-benzo[*d*]imidazol-4-yl)oxy)ethan-1-aminium chloride (**11**) (159 mg, 0.5 mmol), triethylamine (226 mg, 312 ml, 2.25 mmol), NaBH_3_CN (70 mg, 1.125 mmol) in 4 mL of a mixture of THF/MeOH (1:1). Yield: 86.5 mg (49%), white solid. ^1^H NMR (500 MHz, CDCl_3_) δ 7.26–7.33 (m, 8H), 7.20–7.25 (m, 3H), 6.86 (t, *J* = 8.31 Hz, 1H), 6.60 (d, *J* = 8.59 Hz, 1H), 6.49 (d, *J* = 8.02 Hz, 1H), 5.02 (s, 2H), 4.17 (t, *J* = 4.87 Hz, 2H), 3.45 (s, 2H), 3.02 (t, *J* = 4.87 Hz, 2H), 2.85 (d, *J* = 11.46 Hz, 2H), 2.57 (d, *J* = 6.87 Hz, 2H), 1.84–1.96 (m, 2H), 1.69 (d, *J* = 12.03 Hz, 2H), 1.44–1.56 (m, 1H), 1.19–1.33 (m, 3H). ^13^C NMR (126 MHz, CDCl_3_) δ 155.0, 143.2, 138.5, 136.4, 131.4, 129.2 (2C), 128.7 (2C), 128.1 (2C), 127.4 (2C), 126.8 (2C), 121.4, 118.0, 106.7, 102.4, 68.6, 63.4, 55.6, 53.5, 48.9 (2C), 44.6, 36.0 (2C), 30.5. Formula: C_29_H_34_N_4_O_2_; LC-MS: *m/z* 471 (M + H^+^).

##### 1-Benzyl-4-(2-((2-(1-benzylpiperidin-4-yl)ethyl)amino)ethoxy)-1,3-dihydro-2*H*-benzo[*d*]imidazol-2-one (**18**)

Following GP4, compound **18** was prepared using 2-(1-benzylpiperidin-4-yl)acetaldehyde (**7**) (77 mg, 0.375 mmol), 2-((1-benzyl-2-oxo-2,3-dihydro-1*H*-benzo[*d*]imidazol-4-yl)oxy)ethan-1-aminium chloride (**11**) (159 mg, 0.5 mmol), triethylamine (226 mg, 312 mL, 2.25 mmol), NaBH_3_CN (70 mg, 1.125 mmol) in 4 mL of a mixture of THF/MeOH (1:1). Yield: 90 mg (50%), white solid. ^1^H NMR (500 MHz, CDCl_3_) δ 7.33–7.19 (m, 11H), 6.88–6.83 (m, 1H), 6.60 (d, *J* = 8.0 Hz, 1H), 6.49 (d, *J* = 8.0 Hz, 1H), 5.02 (s, 2H), 4.17 (t, *J* = 5.2 Hz, 2H), 3.44 (s, 2H), 3.03 (t, *J* = 5.2 Hz, 2H), 2.84–2.78 (m, 2H), 2.73–2.67 (m, 2H), 1.90–1.83 (m, 2H), 1.57 (br d, *J* = 10.3 Hz, 2H), 1.49–1.44 (m, 2H), 1.30–1.18 (m, 3H) (N*H-*not detected). ^13^C NMR (126 MHz, CDCl_3_) δ 155.1, 143.2, 138.5, 136.4, 131.4, 129.2 (2C), 128.7 (2C), 128.1 (2C), 127.4 (2C), 126.8 (2C), 121.4, 118.1, 106.5, 102.4, 68.5, 63.5, 53.8, 48.7 (2C), 47.1, 44.7, 36.7 (2C), 33.7, 32.3. Formula: C_30_H_36_N_4_O_2_; LC-MS: *m/z* 485 (M + H^+^).

##### 1-Benzyl-4-(2-((3-(1-benzylpiperidin-4-yl)propyl)amino)ethoxy)-1,3-dihydro-2*H*-benzo[*d*]imidazol-2-one (**19**)

Following GP4, compound **19** was prepared using 3-(1-benzylpiperidin-4-yl)propanal (**8**) (87 mg, 0.375 mmol), 2-((1-benzyl-2-oxo-2,3-dihydro-1*H*-benzo[*d*]imidazol-4-yl) oxy)ethan-1-aminium chloride (**11**) (159 mg, 0.5 mmol), triethylamine (226 mg, 312 ml, 2.25 mmol), NaBH_3_CN (70 mg, 1.125 mmol) in 4 mL of a mixture of THF/MeOH (1:1). Yield: 97 mg (52%), white solid. ^1^H NMR (500 MHz, CDCl_3_) δ 7.30–7.26 (m, 9H), 7.25–7.20 (m, 3H), 6.86 (t, *J* = 8.0 Hz, 1H), 6.60 (d, *J* = 8.0 Hz, 1H), 6.49 (d, *J* = 7.4 Hz, 1H), 5.01 (s, 2H), 4.18 (t, *J* = 4.9 Hz, 2H), 3.46 (s, 2H), 3.04 (t, *J* = 4.9 Hz, 2H), 2.83 (br d, *J* = 12.0 Hz, 3H), 2.72–2.64 (m, 2H), 1.87 (br t, *J* = 10.9 Hz, 2H), 1.63–1.50 (m, 4H), 1.23–1.18 (m, 4H). ^13^C NMR (126 MHz, CDCl_3_) δ 155.0, 143.1, 138.5, 136.4, 131.4, 129.3 (2C), 128.7 (2C), 128.1 (2C), 127.6, 127.3 (2C), 126.8, 121.5, 118.1, 106.8, 102.5, 99.9, 63.5, 53.8 (2C), 49.8, 48.6, 44.6, 35.5, 34.1, 32.3 (2C), 26.9. Formula: C_31_H_38_N_4_O_2_; LC-MS: *m/z* 499 (M + H^+^).

### 3.2. Molecular Modelling

All simulations, analyses and visualisations were conducted with Maestro software (Schrödinger LLC, New York, NY, 2020-4 Release).

Molecules underwent energy minimisation with LigPrep script and the most viable conformations were used in docking studies with Glide script. Molecular modelling was performed with the OPLS_2005 force field. Crystal structure of BuChE was extracted from PDB (ID: 7awh) and refined with Protein Preparation Wizard script, no constraints were applied in the docking process. The homology model of 5-HT_6_ used in docking studies was developed with well-validated method and successfully utilised in previously published studies [38]. The only grid-based constraint applied in the docking processes was H-bond formation with Asp3.32.

To investigate activity differences in BuChE between related molecules (12, 14, 16, 19), the MD simulations were conducted with Desmond MD System (D. E. Shaw Research, New York, NY, 2020, Maestro-Desmond Interoperability Tools, Schrödinger, New York, NY, 2020-4 Release). Systems were solvated in 10Å cubic box, TIP3P solvent model in 0.15 mM KCl and neutralised by the addition of 6 Cl^−^ ions, with OPLS_2005 force field. The default settings were applied for MD simulations; the time of simulation was 10 ns. Interactions lasting more than 30% of the simulation time were documented.

### 3.3. Radioligand Binding Assay

#### 3.3.1. Preparation of Solutions of Test and Reference Compounds

We prepared 10 mM of stock solutions of tested compounds in DMSO. Serial dilutions of compounds were prepared in a 96-well microplate in assay buffers using the automated pipetting system epMotion 5070 (Eppendorf). Each compound was tested in 8 concentrations from 10^−5^ to 10^−12^ M (final concentration).

#### 3.3.2. 5-HT_6_ Receptor Binding Assay

The radioligand binding assay was performed using membranes from CHO-K1 cells stably transfected with the human 5-HT_6_ receptor (PerkinElmer) according to the previously described procedure [38]. All assays were carried out in duplicates. We prepared 50 µL working solution of the tested compounds, 50 µL [^3^H]-LSD (final concentration 2.5 nM) and 150 µL diluted membranes (15 µg protein per well) in assay buffer (50 mM Tris, pH 7.4, 10 mM MgCl_2_, 0.1 mM EDTA) and transferred them to polypropylene 96-well microplates using a 96-well pipetting station Rainin Liquidator (MettlerToledo). Methiothepin (10 μM) was used to define nonspecific binding. The microplate was covered with sealing tape, mixed and incubated for 60 min at 27 °C. The reaction was terminated by rapid filtration through GF/B filter mate presoaked with 0.5% polyethyleneimine for 30 min. Ten rapid washes with 200 µL 50 mM Tris buffer (4 °C, pH 7.4) were performed using the automated harvester system Harvester-96 MACH III FM (Tomtec). The filter mates were dried at 37 °C in a forced air fan incubator and then solid scintillator MeltiLex was melted on filter mates at 90 °C for 5 min. Radioactivity was counted in MicroBeta2 scintillation counter (PerkinElmer). Data were fitted to a one-site curve-fitting equation with Prism 6 (GraphPad Software) and *K*_i_ values were estimated from the Cheng−Prusoff equation.

### 3.4. Human Acetylcholinesterase and Butyrylcholinesterase In Vitro Inhibitory Activity

The assay was based on spectrophotometric Ellman’s method [49] and was performed in 96-well microplates with the reagents purchased from Sigma-Aldrich (Steinheim, Germany) according to the previously described procedure [38]. *h*BuChE was kindly donated by Vivonics (Bedford, MA, USA). Stock solutions of the tested compounds (1.14 mM) and reagents were prepared in DMSO and water, respectively. Before starting the enzymatic reaction, the test compound or water or mixture of DMSO/water in appropriate ratio (0.025 mL) were incubated in a phosphate buffer (0.2 mL, 100 mM, pH 8.0) with Ellman’s reagent (0.02 mL; 2.5 mM) and *h*AChe or *h*BuChE (0.02 mL; 0.384 U/mL) at 36 °C for 5 min. Then, substrate solutions (0.02 mL, 3.75 mM) were added: acetylthiocholine for *h*AChE or butyrylthiocholine for *h*BuChE. After 5 minutes of the enzymatic reaction, the changes in absorbance were measured at 412 nm, using a microplate reader (SPECTROstar Nano; BMG Labtech, Ortenberg, Germany). First, compounds were tested at the screening concentration of 10 µM. Based on the equation 100 − (S/B) × 100 (where S and B were the respective enzyme activities with and without the test sample, respectively) the percentage inhibition of each enzyme for each compound was calculated. Next, compounds with enzyme inhibitory activity higher than 50% were tested again at seven different concentrations to determine their IC_50_ values. The IC_50_ values were calculated using nonlinear regression (GraphPad Prism 5; GraphPad Software, San Diego, CA, USA) by plotting the residual enzyme activities against the applied inhibitor concentration. Donepezil and tacrine were used as the reference compounds. All experiments were performed in triplicate.

### 3.5. Free Radical Scavenging In Vitro Activity

The spectrophotometric ABTS assay was performed in 96-well microplates with the reagents purchased from Sigma-Aldrich (Steinheim, Germany) according to the previously described procedure [54]. Stock solutions of the tested compounds (6.4 mM) and reagents were prepared in DMSO and water, respectively. 2,2′-Azino-bis(3-ethylbenzothiazoline-6-sulfonic acid) free radical (ABTS^•^) was prepared by mixing the stock solution of ABTS (7 mM) and K_2_S_2_O_3_ (2.45 mM) in ratio 1:1. The mixture was kept in the dark at 4 °C for 16 h. The obtained solution of (ABTS^•^) was diluted with water 20 times. Then, the test compound or water or mixture of DMSO/water in the appropriate ratio (0.02 mL) was added to ABTS^•^ solution (0.18 mL). The three concentrations of the test compounds and Trolox were tested in triplicate: 0.15, 0.075 and 0.03 mM. Changes in absorbance were measured at 734 nm after 5, 30 and 60 min, using a microplate reader (SPECTROstar Nano; BMG Labtech, Ortenberg, Germany). Based on the equation ((A_0_ − A)/A_0_) × 100% (where A_0_ and A were the absorbances without and with the test compound, respectively) the percentage of free radical scavenging activity was calculated. The results are presented as mean ± SD.

### 3.6. Metal-Chelating In Vitro Activity

The spectrophotometric assessment of metal-chelating properties was performed in UV-transparent 96-well microplates (Corning^®^ Costar) with the reagents purchased from Sigma-Aldrich (Steinheim, Germany) according to the previously published procedure [54]. Stock solutions of the tested compounds (6.4 mM) were prepared in DMSO and diluted with water to the final concentration of 0.5 mM. Stock solutions of cation salts (0.5 mM)—ZnCl_2_, CoCl_2_, AlCl_3_, NiCl_2_, CaCl_2_, MgCl_2_, Pb(CH_3_COO)_2_, FeSO_4_, FeCl_3_—were prepared in water. The test compound, or the cation salt or water or mixture of DMSO/water in the appropriate ratio (0.02 mL), or the mixture of the test compound and the cation salt (0.04 mL, ratio 1:1) were incubated in HEPES buffer (0.18 mL or 0.16 mL for the mixture of compound and the cation salt, 20 mM, pH 7.4) at room temperature for 30 min. Then, absorption spectra in the range of 250–400 nm (4 nm interval) were recorded using a microplate reader (SPECTROstar Nano; BMG Labtech, Ortenberg, Germany). A shift of absorbance was calculated as differences between the absorbance of the mixture of the compound and the cation salt and the sum of the absorbances of the compound and the cation salt.

### 3.7. ADME-Tox Parameters

The ADME-Tox parameters were determined in vitro as described previously [61]. Statistical significances were analysed by GraphPad Prism^™^ 8 software using one-way ANOVA and Bonferroni’s multiple comparison post-test. All reference compounds (caffeine, doxorubicin, ketoconazole, quinidine and verapamil) were purchased from Sigma-Aldrich (St. Louis, MO, USA). All UPLC/MS analyses were done by Waters ACQUITY TQD system with the TQ Detector (Waters, Milford, USA). The absorbance and luminescence were measured using a microplate reader (EnSpire Multimode; PerkinElmer, Waltham, MA, USA).

#### 3.7.1. Metabolic Stability in Human Liver Microsomes

The metabolic stability was evaluated by incubation of compounds with human liver microsomes (Sigma-Aldrich, St. Louis, MO, USA) in 10 mM Tris–HCl buffer (pH 7.4) at 37 °C for 120 min in the presence of NADPH Regeneration System (Promega, Madison, WI, USA). The percentage of the remaining substrate and the most probable metabolic pathways were estimated by UPLC-MS analyses of the reaction mixtures.

#### 3.7.2. Influence on CYP P450 Activity

The influence on CYP 3A4 and 2D6 activity was estimated by respective luminescent CYP P450-Glo™ assay (Promega^®^, Madison, WI, USA). The compounds were tested in the concentration range of 0.1–25 µM. The results were compared to the respective reference CYP inhibitors: ketoconazole and quinidine.

#### 3.7.3. Hepatotoxicity in HepG2 Cells

The HepG2 (ATCC^®^ HB-8065™) cell line was cultivated according to the procedure provided by ATCC. The tested compounds were incubated with cells for 72 h in the following concentrations: 1, 10, 50 and 100 μM. To evaluate the cells’ viability, CellTiter 96^®^ AQ_ueous_ Non-Radioactive Cell Proliferation Assay (MTS, Promega^®^, Madison, WI, USA) was performed. The antiproliferative drug doxorubicin was used as a positive control at the concentration of 1 µM. For determination of the IC_50_ value for compound **14,** additional study was performed. The tested compound was incubated with the HepG2 cells for 72 h in the following nine concentrations: 0.001, 0.01, 0.1, 1, 5, 10, 25, 50 and 100 μM. The cells’ viability was estimated by MTS test. IC_50_ was calculated by Graph Pad Prism 8.0.1 software.

## 4. Conclusions

Therapeutic options in AD treatment are limited to drugs that temporarily relieve the symptoms of the disease—cholinesterase inhibitors (donepezil, rivastigmine and galantamine) and NMDA receptor antagonists (memantine). In 2021 the U.S. Food and Drug Administration approved aducanumab—an amyloid beta-directed monoclonal antibody—the first drug with disease-modifying potential. Aducanumab was approved under the accelerated approval pathway, based on effects on a surrogate endpoint, which in this case was a reduction of amyloid beta plaques in the brains of patients with AD. Its efficacy in terms of clinical benefits is yet to be determined [62,63]. Hence, there is still an unmet need to search for effective therapy for AD, primarily with disease-modifying effects but preferably with a symptoms-relieving component for patients with the already developed disease. Following this idea, we have been working on multifunctional ligands that address the neurotransmission disorders and oxidative stress underlying AD. Within the synthesised and evaluated compounds, we selected compound **14,** with an excellent and balanced potency against *h*5-HT_6_R (*K*_i_ = 22 nM) and *h*BuChE (IC_50_ = 16 nM) and free radicals scavenging activity. The compound is metabolically stable both on human and mouse liver microsomes and safe in the concentrations required for modulation of *h*5-HT_6_R and *h*BuChE. Therefore, compound **14** may serve as a promising candidate for further development of multitarget-directed ligands aiming at symptoms and causes of Alzheimer’s disease.

## Figures and Tables

**Table 1:** Affinity for 5-HT_6_R and inhibition of *h*AChE and *h*BuChE by compounds **12**–**19**.

Cmpd.	n	*h*5-HT_6_R *K*_i_ (µM) ^a^	*h*AChE IC_50_ (µM) ^b^	*h*BuChE IC_50_ (µM) ^b^
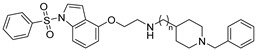				
**12**	1	0.021 ± 0.001	3.151 ± 0.081	2.325 ± 0.059
**13**	2	0.017 ± 0.002	2.265 ± 0.091	1.115 ± 0.031
**14**	3	0.022 ± 0.002	0.930 ± 0.035	0.016 ± 0.001
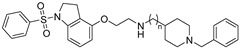				
**15**	1	0.267 ± 0.009	8.207 ± 0.225	5.405 ± 0.100
**16**	3	0.598 ± 0.019	0.821 ± 0.014	0.487 ± 0.012
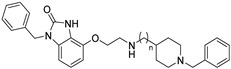				
**17**	1	0.136 ± 0.022	39.5% ± 1.3 ^c^	44.3% ± 2.0 ^c^
**18**	2	0.142 ± 0.020	2.856 ± 0.068	1.158 ± 0.024
**19**	3	0.480 ± 0.048	0.544 ± 0.015	0.613 ± 0.011
**Donepezil**		-	0.006 ± 0.0001	-
**Tacrine**		-	0.131 ± 0.002	0.034 ± 0.0004
**Mianserin**		0.056 ± 0.012	-	-

^a^*K*_i_ values are expressed as the mean ± standard error of the mean (SEM) of at least three experiments. ^b^ IC_50_ values are expressed as the mean ± standard error of the mean (SEM) of at least three experiments. ^c^ % of inhibition of the enzymes observed at 10 µM concentration of **17**. *h*5-HT_6_R-recombinant human 5-HT_6_ receptors, *h*AChE-human recombinant AChE, *h*BuChE-human recombinant BuChE.

**Table 2:** The results of metabolic stability evaluation on human liver microsomes (HLMs) and mouse liver microsomes (MLMs) for compounds **14**, **16**, **19** and verapamil (control). * most probable metabolic pathways were estimated by MS analyses supported by in silico prediction using MetaSite 8.0.1; main metabolic pathways are bolded.

		HLMs			MLMs		
Comp.	(*m/z*)	% Left	Metabolite (*m/z*)	Metabolic Pathway *	% Left	Metabolite (*m/z*)	Metabolic Pathway *
**14**	532.35	**84.69**	317.23 (**M1**)	**ox. deamination**	**75.54**	317.23 (**M1**)	**ox. deamination**
			442.30 (M2)	ox. deamination		442.37 (M2)	ox. deamination
**16**	534.41	**84.40**	444.30 (**M1**)	**ox. deamination**	**84.48**	444.36 (**M1**)	**ox. deamination**
			550.37 (M2)	hydroxylation		550.56 (M2)	hydroxylation
**19**	499.39	**94.00**	409.41 (**M1**)	**ox. deamination**	**79.38**	409.41 (**M1**)	**ox. deamination**
						284.27 (M2)	ox. deamination
**Verapamil**	455.31	**30.84**	441.35 (**M1**)	**demethylation**	**23.93**	441.42 (**M1**)	**demethylation**
			291.28 (M2)	decomposition		441.42 (M2)	demethylation
			165.09 (M3)	decomposition		291.35 (M3)	decomposition
			441.29 (M4)	demethylation		293.34 (M4)	decomp./hydrox.
			427.33 (M5)	double demethylation		277.33 (M5)	decomposition
			277.26 (M6)	decomposition			

## Supplementary Materials

The following supporting information can be downloaded at: https://www.mdpi.com/article/10.3390/ijms23169443/s1.
