# Supplementary material for: Serotonin 5-HT6 Receptor Ligands and Butyrylcholinesterase Inhibitors Displaying Antioxidant Activity—Design, Synthesis and Biological Evaluation of Multifunctional Agents against Alzheimer’s Disease
Source: Int J Mol Sci. 2022 Aug 21;23(16):9443. doi: 10.3390/ijms23169443 (PMC9409043; doi:10.3390/ijms23169443)
Supplement: Supplementary file 1 [file ijms-23-09443-s001.zip › ijms-1882341-supplementary.pdf]

Article

Serotonin 5-HT6 Receptor Ligands and Butyrylcholinesterase Inhibitors Displaying Antioxidant Activity—Design, Synthesis and Biological Evaluation of Multifunctional Agents against Alzheimer's Disease

Krzysztof Więckowski [1], Natalia Szałaj [2], Beata Gryzło [1], Tomasz Wichur [2], Izabella Góral [2,3], Emilia Sługocka [2,3], Joanna Sniecikowska [1], Gniewomir Latacz [4], Agata Siwek [5], Justyna Godyń [2], Adam Bucki [1], Marcin Kołaczkowski [1] and Anna Więckowska [2,*]

[1] Department of Medicinal Chemistry, Faculty of Pharmacy, Jagiellonian University Medical College, 9 Medyczna St., 30-688 Kraków, Poland
[2] Department of Physicochemical Drug Analysis, Faculty of Pharmacy, Jagiellonian University Medical College, 9 Medyczna St., 30-688 Kraków, Poland
[3] Doctoral School of Medical and Health Sciences, Jagiellonian University Medical College, 16 Łazarza St., 31-530 Kraków, Poland
[4] Department of Technology and Biotechnology of Drugs, Faculty of Pharmacy, Jagiellonian University Medical College, 9 Medyczna St., 30-688 Kraków, Poland
[5] Department of Pharmacobiology, Faculty of Pharmacy Jagiellonian University Medical College, 9 Medyczna St., 30-688 Kraków, Poland
* Correspondence: anna.wieckowska@uj.edu.pl

Table of contents

1. Antioxidant activity 2
2. Metabolic stability – human liver microsomes 3
3. Metabolic stability – mouse liver microsomes 9

1. Antioxidant activity

Table S1. Results of ABTS assay: free radical scavenging potency of the tested compounds and Trolox (at 150 µM, 75 µM and 30 µM after 5, 30 and 60 min) as an [%] of ABTS• reduction ± SD.

**Compd.**	**Conc. (µM)**	**% of ABTS• reduction ± SD**		
		**5 min**	**30 min**	**60 min**
**12**	150	20±3	30±3	39±4
	75	13±0	19±0	24±1
	30	8±1	9±0	10±0
**13**	150	20±0	29±1	34±1
	75	9±1	11±1	13±1
	30	5±1	5±1	6±1
**14**	150	20±0	29±0	35±0
	75	13±1	18±0	21±0
	30	5±3	5±3	6±3
**15**	150	19±1	29±1	36±4
	75	11±1	13±1	15±1
	30	10±0	10±1	11±0
**16**	150	10±0	19±1	24±1
	75	7±2	9±2	11±3
	30	7±0	7±1	7±1
**17**	150	19±2	32±2	39±2
	75	12±2	22±2	27±1
	30	8±2	14±3	18±4
**18**	150	13±0	33±0	40±0
	75	11±1	31±1	36±2
	30	10±1	23±1	27±1
**19**	150	25±4	37±5	43±4
	75	14±1	24±2	30±2
	30	10±0	17±0	22±1
**Trolox**	150	100±0	100±0	100±0
	75	100±0	100±0	100±

	30	59±2	59±2	59±2

2. Metabolic stability – human liver microsomes

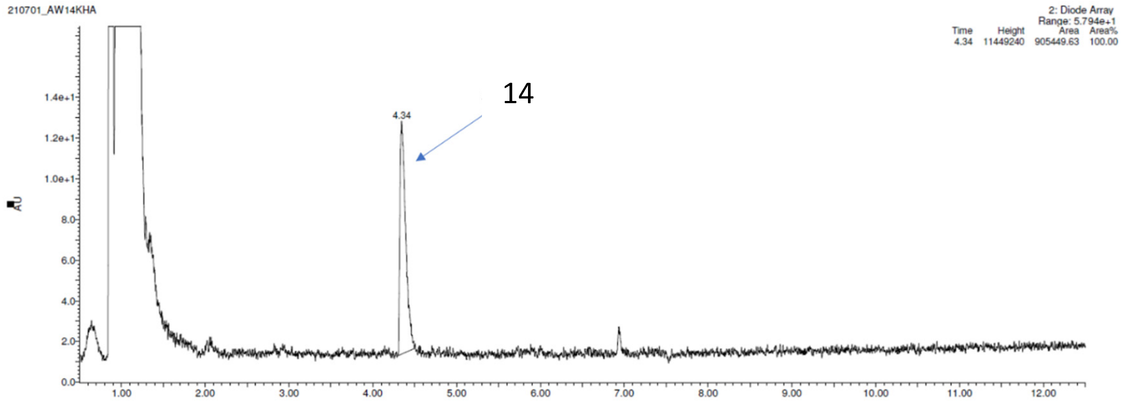

Figure S1. UPLC of control reaction (120 min incubation of compound **14** in the reaction buffer without microsomes).

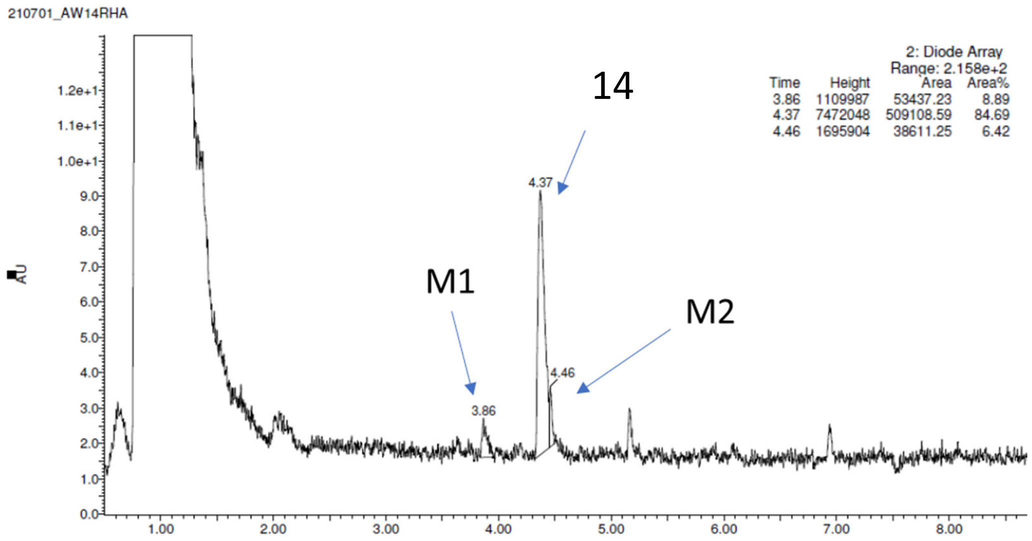

Figure S2. UPLC after 120 min incubation of compound **14** with HLMs.

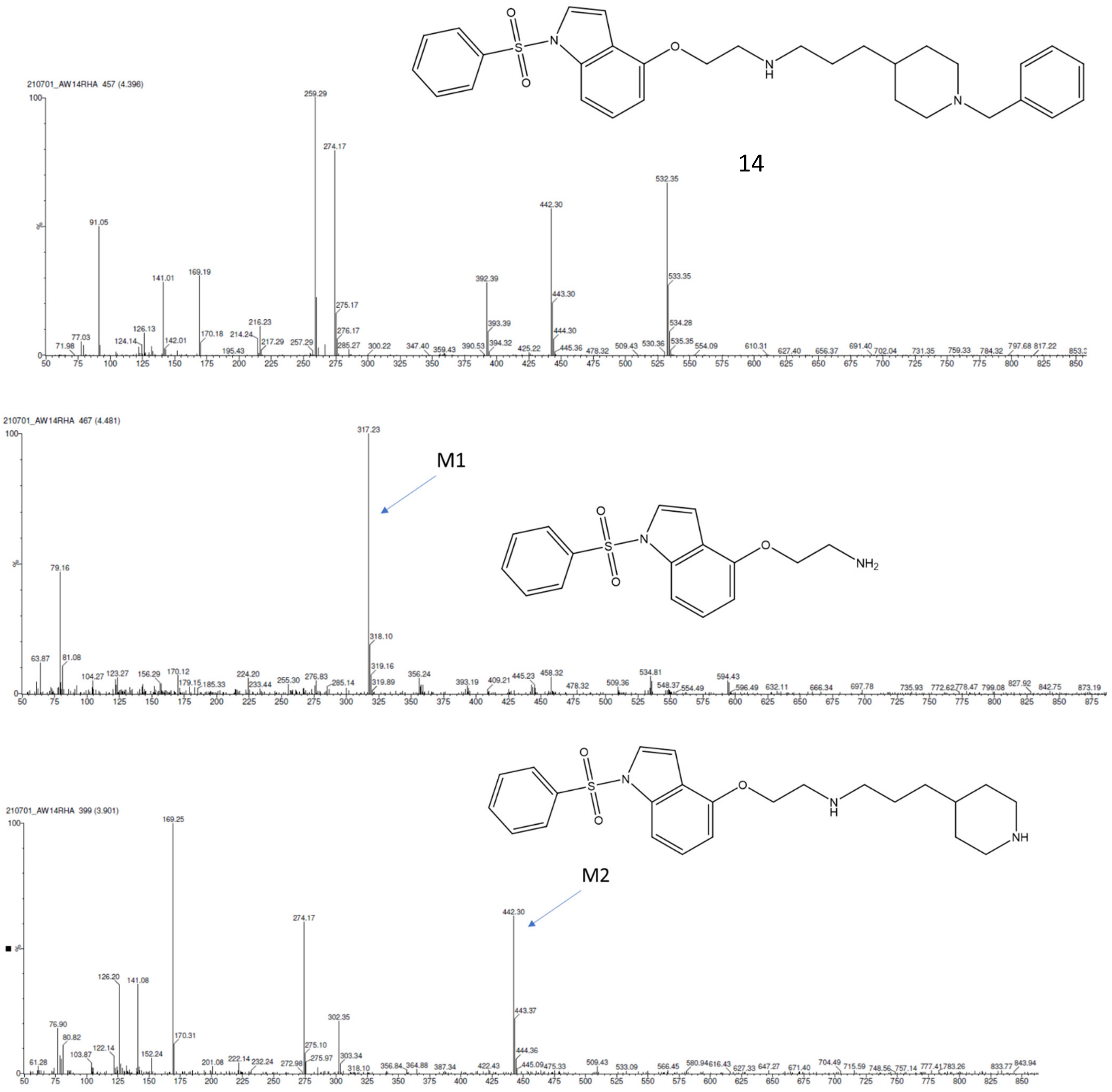

Figure S3. MS analyses of **14** and its metabolites.

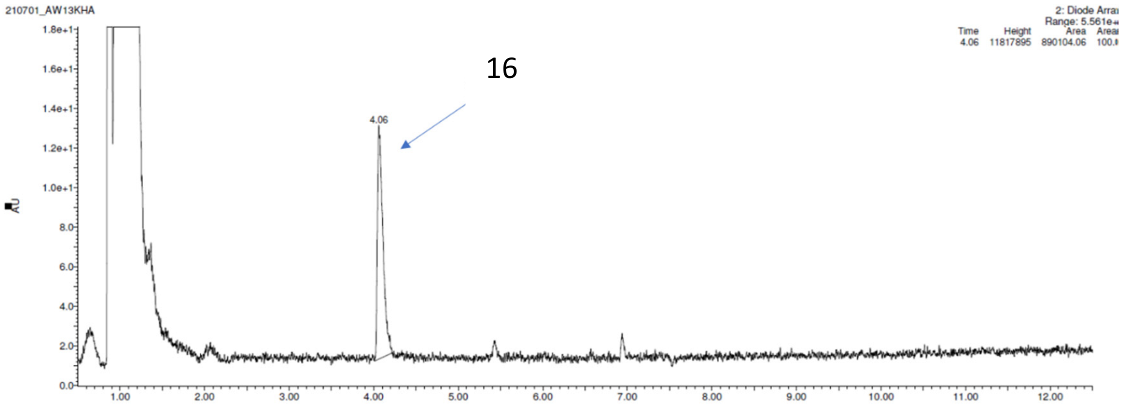

Figure S4. UPLC of control reaction (120 min incubation of compound **16** in the reaction buffer without microsomes).

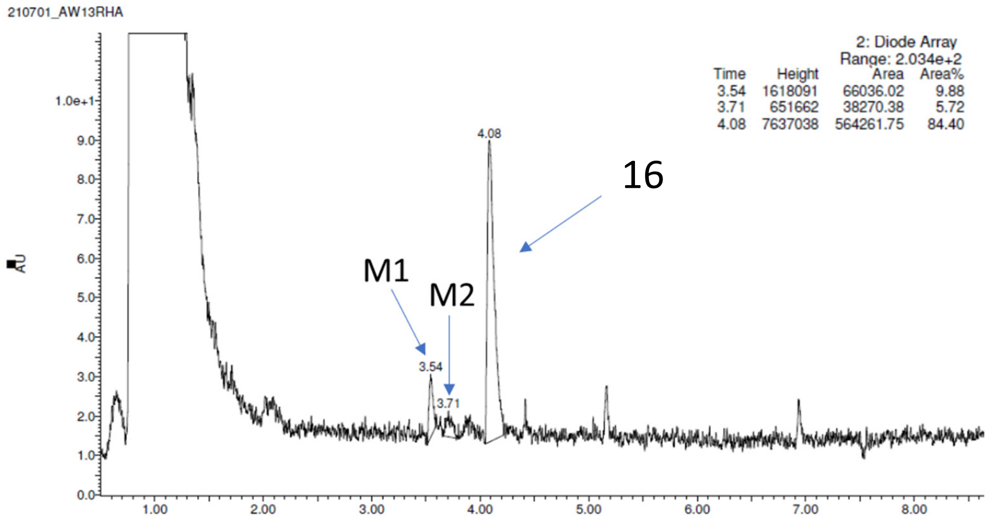

Figure S5. UPLC after 120 min incubation of compound **16** with HLMs.

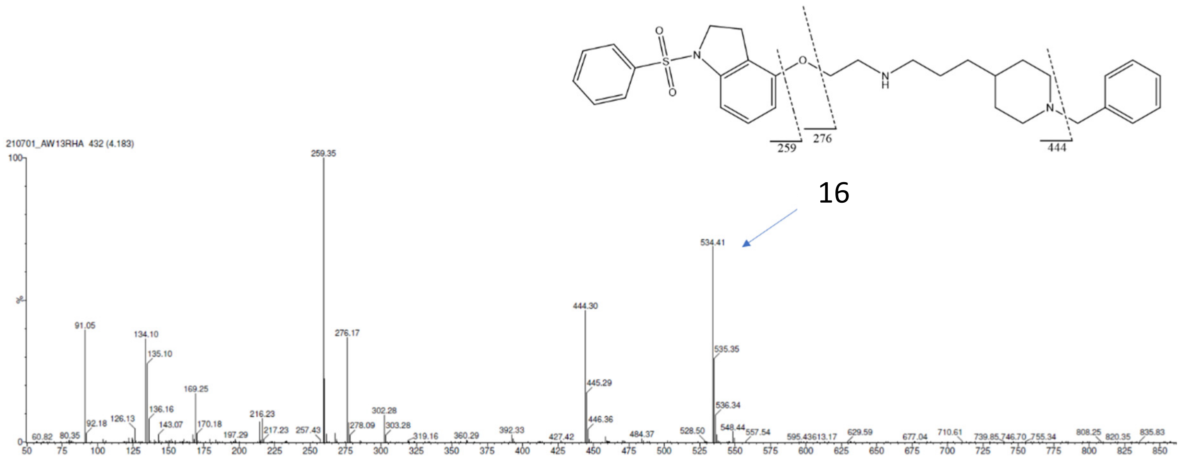

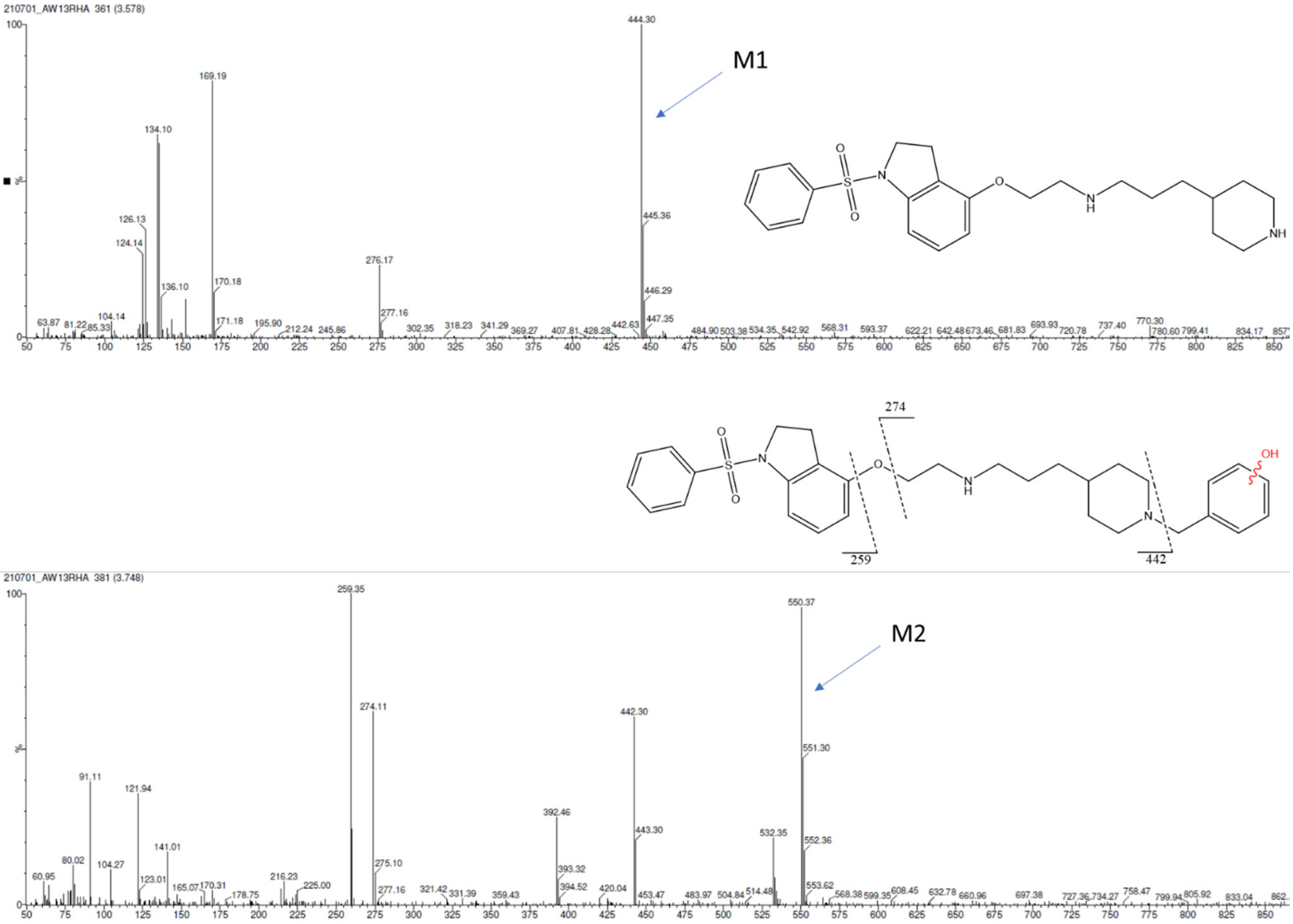

Figure S6. MS analyses of **16** and its metabolites.

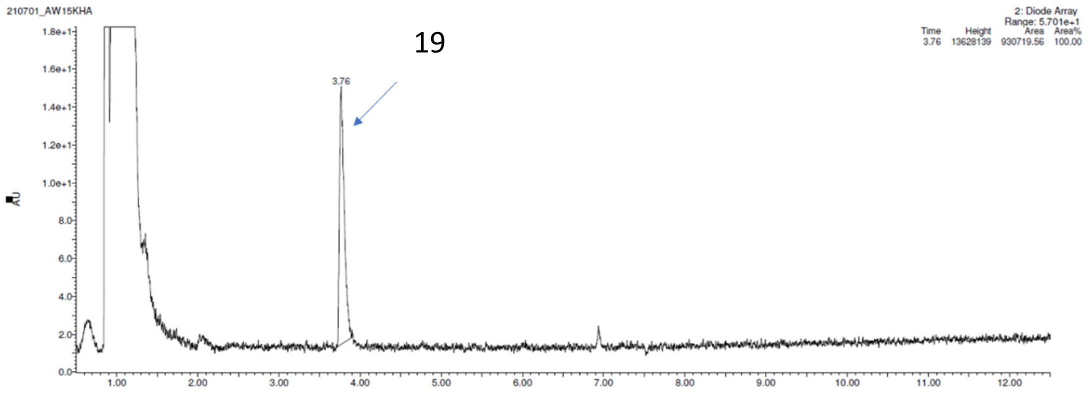

Figure S7. UPLC of control reaction (120 min incubation of compound **19** in the reaction buffer without microsomes).

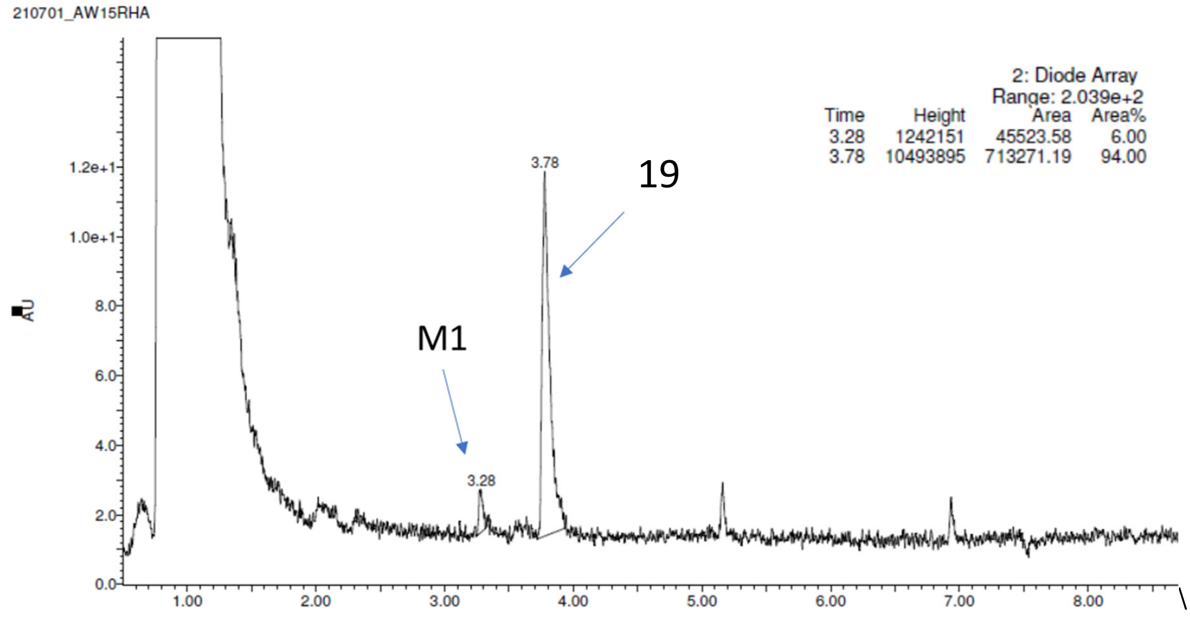

Figure S8. UPLC after 120 min incubation of compound **19** with HLMs.

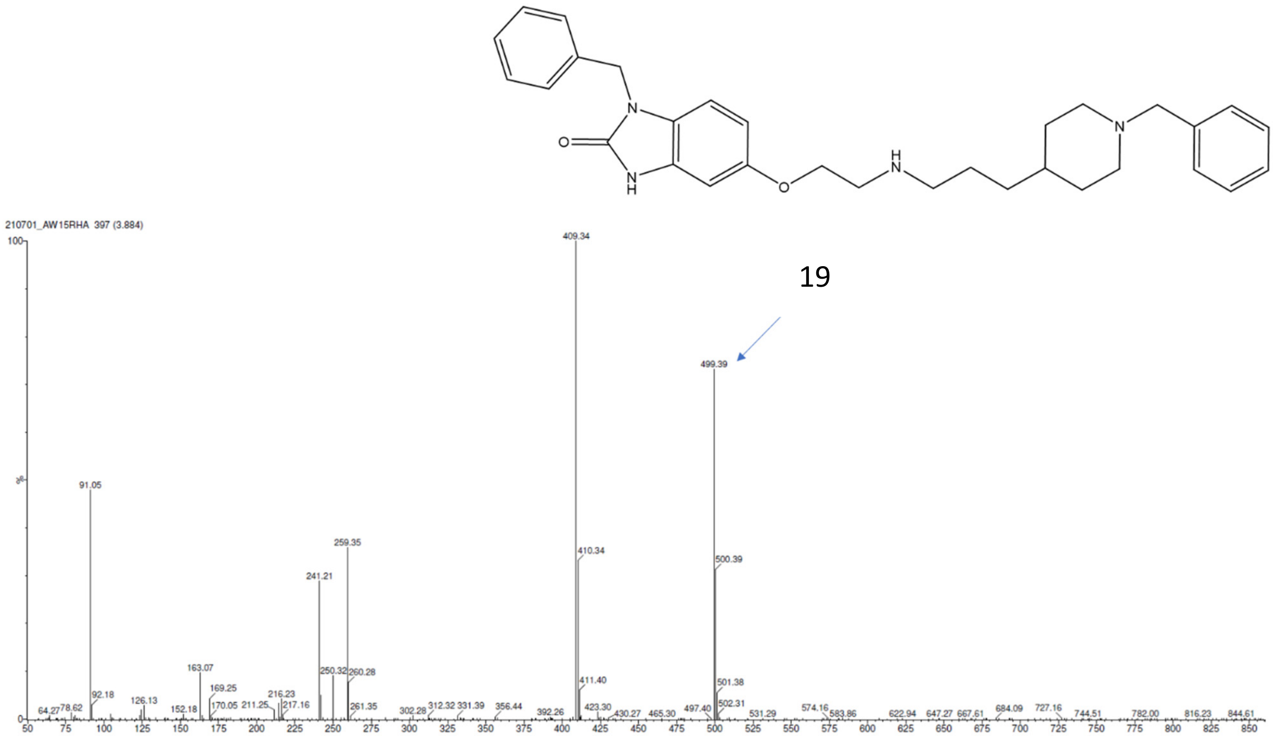

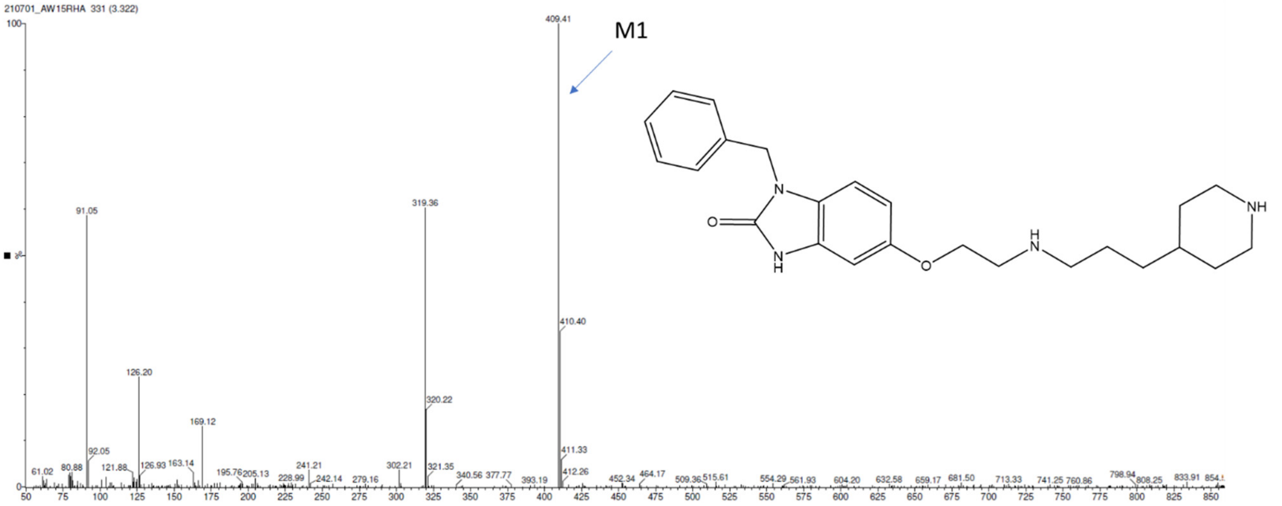

Figure S9. MS analyses of **19** and its metabolites.

3. Metabolic stability – mouse liver microsomes

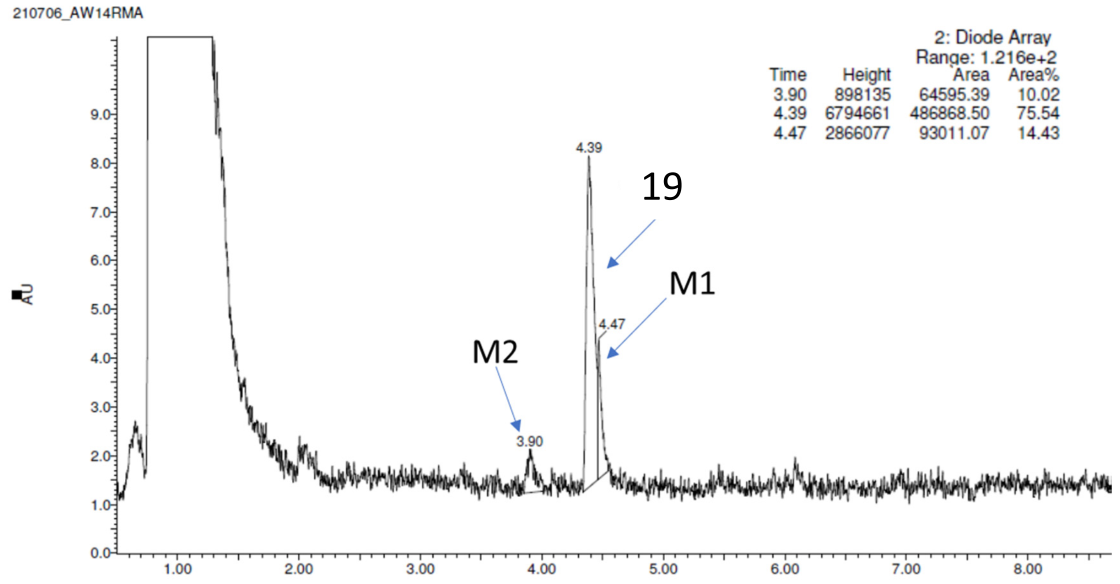

Figure S10. UPLC after 120 min incubation of compound **14** with MLMs.

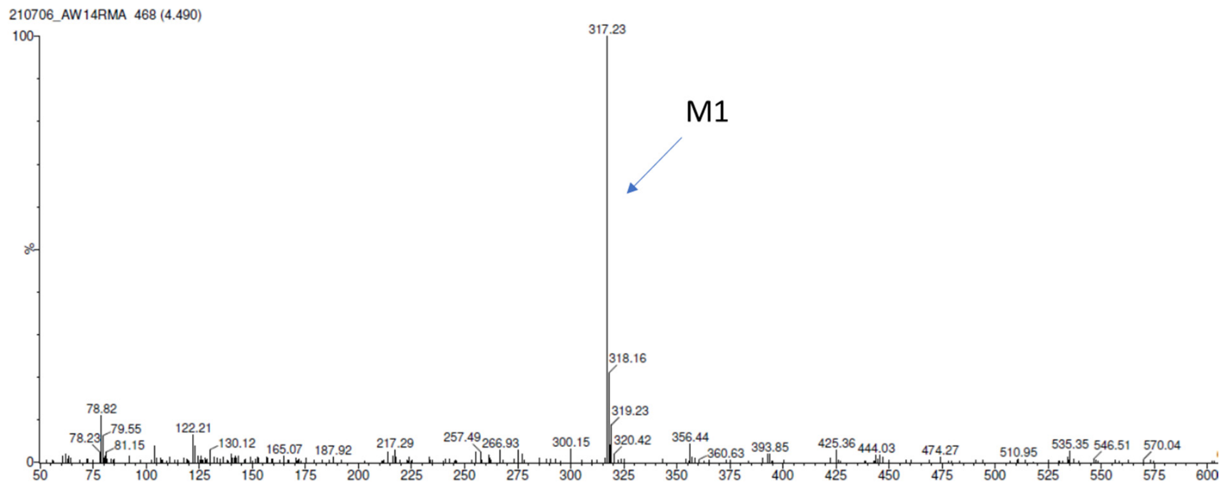

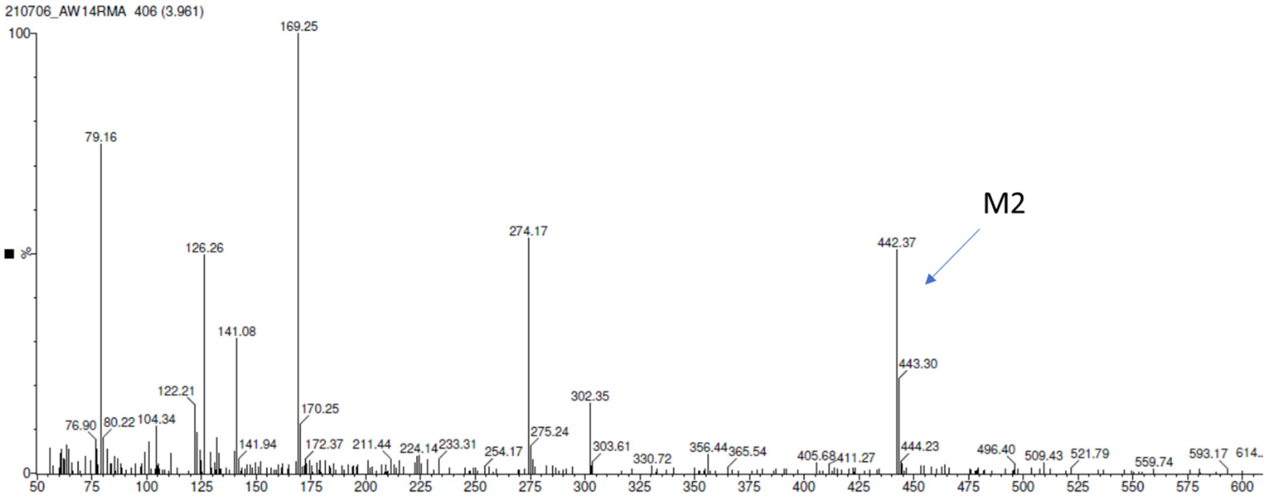

Figure S11. MS analyses of **14** metabolites present after incubation with MLMs.

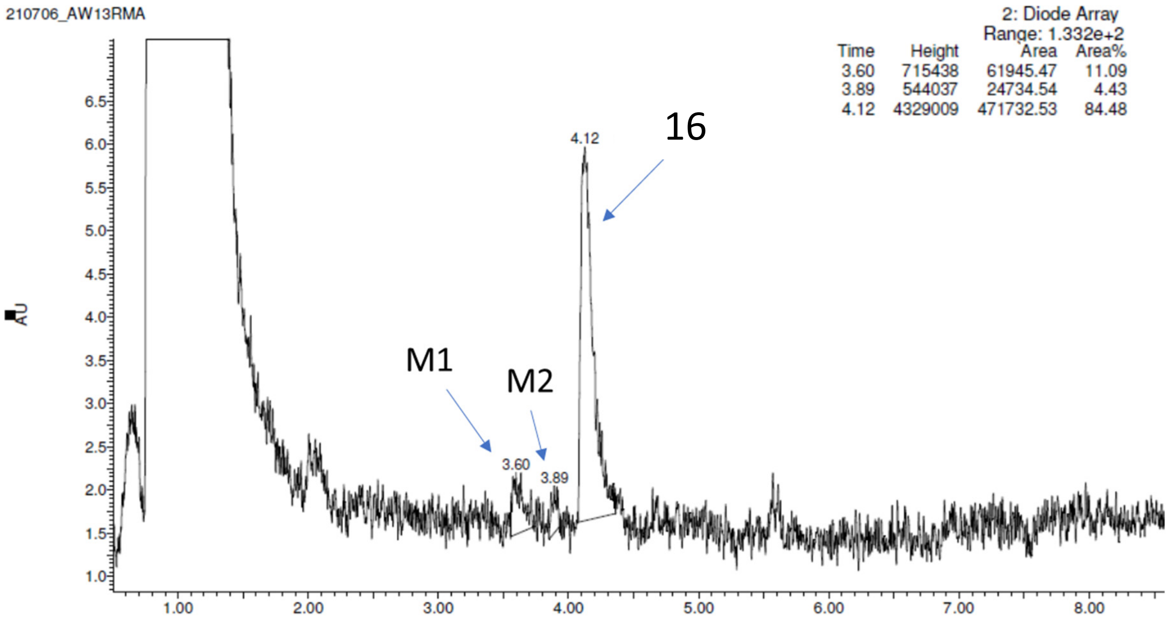

Figure S12. UPLC after 120 min incubation of compound **16** with MLMs.

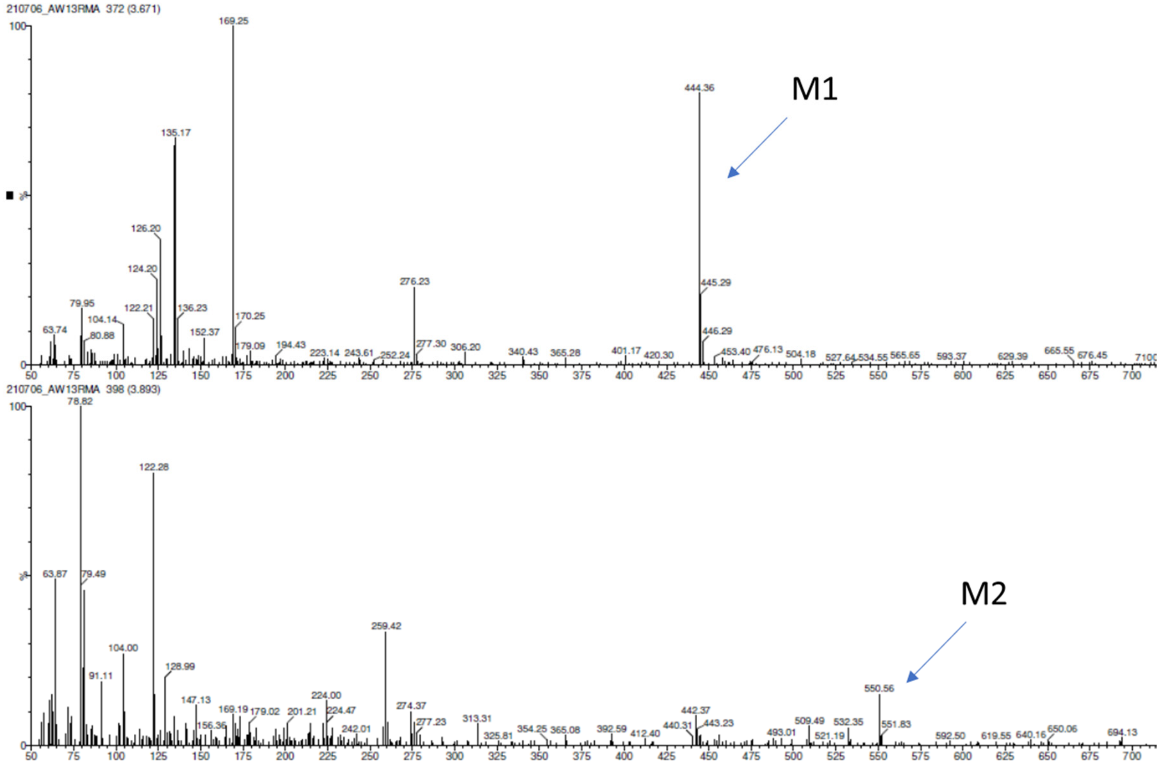

Figure S13. MS analyses of **16** metabolites present after incubation with MLMs.

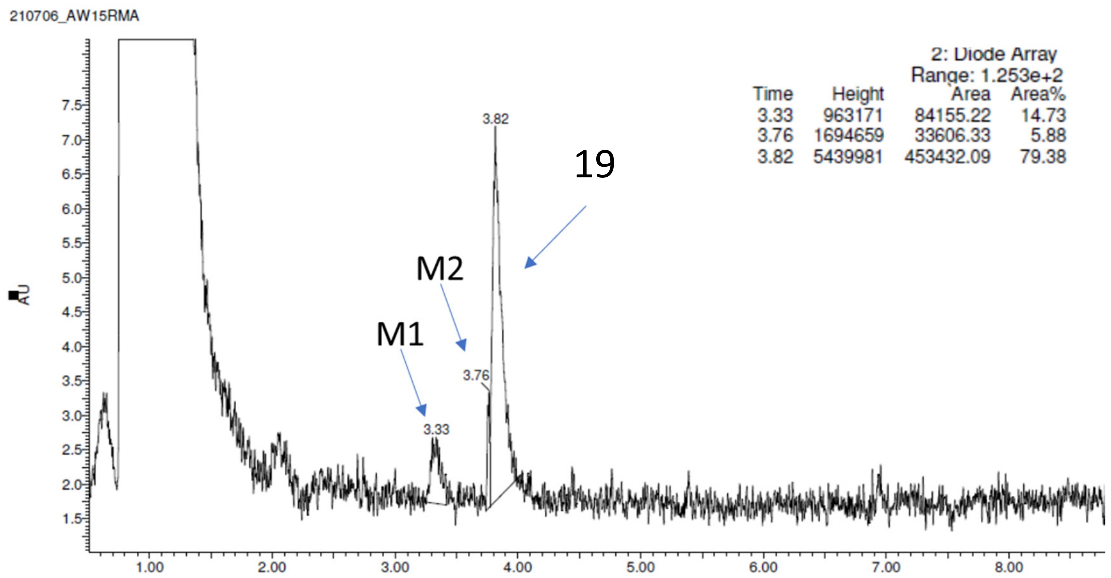

Figure S14. UPLC after 120 min incubation of compound **19** with MLMs.

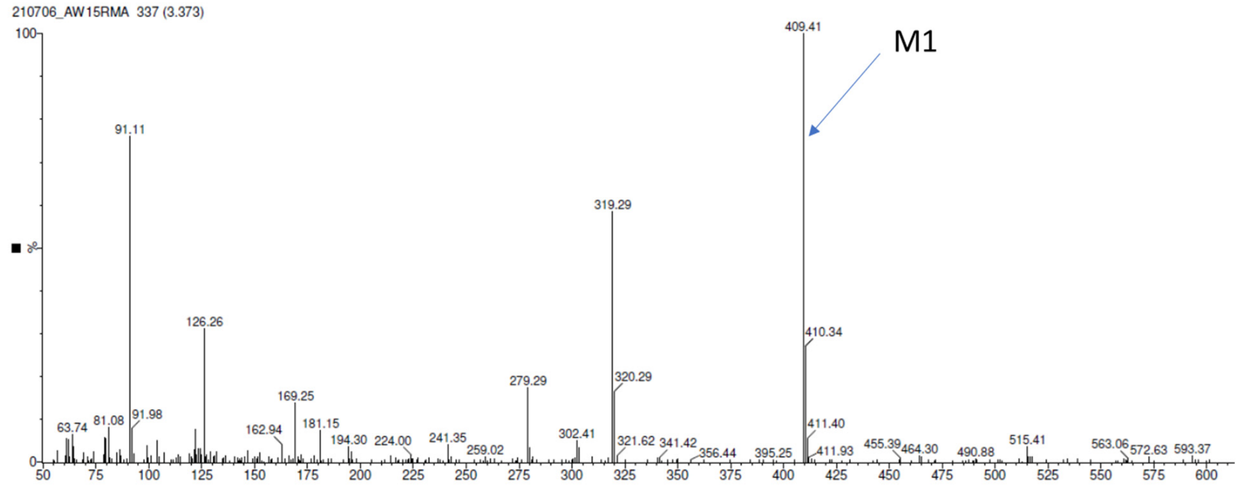

Figure S15. MS analysis of **19** metabolite M1 present after incubation with MLMs.

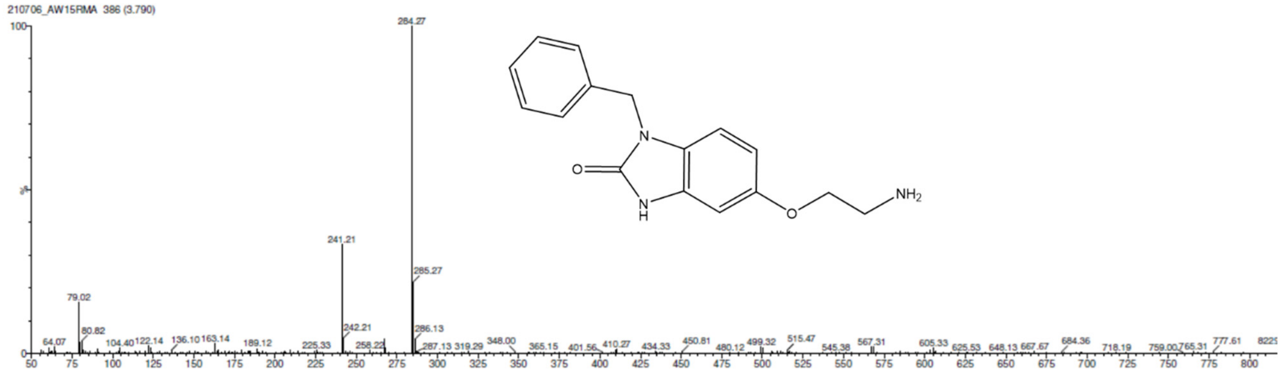

Figure S16. MS analysis of **19** metabolite M2 present **only** after incubation with MLMs.
